# ADIPOR1 is essential for vision and its RPE expression is lost in the *Mfrp*^*rd6*^ mouse

**DOI:** 10.1038/s41598-018-32579-9

**Published:** 2018-09-25

**Authors:** Valentin M. Sluch, Angela Banks, Hui Li, Maura A. Crowley, Vanessa Davis, Chuanxi Xiang, Junzheng Yang, John T. Demirs, Joanna Vrouvlianis, Barrett Leehy, Shawn Hanks, Alexandra M. Hyman, Jorge Aranda, Bo Chang, Chad E. Bigelow, Dennis S. Rice

**Affiliations:** 1 Department of Ophthalmology, Novartis Institutes for BioMedical Research, Cambridge, Massachusetts, United States; 2 Global Scientific Operations, Novartis Institutes for BioMedical Research, Cambridge, Massachusetts, United States; 3 The Jackson Laboratory, Bar Harbor, Maine, United States

## Abstract

The knockout (KO) of the adiponectin receptor 1 (*AdipoR1*) gene causes retinal degeneration. Here we report that ADIPOR1 protein is primarily found in the eye and brain with little expression in other tissues. Further analysis of *AdipoR1* KO mice revealed that these animals exhibit early visual system abnormalities and are depleted of RHODOPSIN prior to pronounced photoreceptor death. A KO of *AdipoR1* post-development either in photoreceptors or the retinal pigment epithelium (RPE) resulted in decreased expression of retinal proteins, establishing a role for ADIPOR1 in supporting vision in adulthood. Subsequent analysis of the *Mfrp*^*rd6*^ mouse retina demonstrated that these mice are lacking ADIPOR1 in their RPE layer alone, suggesting that loss of ADIPOR1 drives retinal degeneration in this model. Moreover, we found elevated levels of IRBP in both the *AdipoR1* KO and the *Mfrp*^*rd6*^ models. The spatial distribution of IRBP was also abnormal. This dysregulation of IRBP hypothesizes a role for ADIPOR1 in retinoid metabolism.

## Introduction

The adiponectin receptor 1 (*ADIPOR1*) gene encodes a seven transmembrane protein named for its purported cognate ligand adiponectin, a secreted hormone that is inversely correlated with development of obesity, insulin resistance, and type 2 diabetes^1,2^. It has been reported that adiponectin acts through binding to its receptors, adiponectin receptors 1 and 2 (ADIPOR1 and ADIPOR2), respectively^1,2^, and this relationship has been further propagated by studies that have shown that ablation of *AdipoR1*/*AdipoR2* in mice led to the induction of insulin resistance and glucose intolerance^3^. However, a contemporaneous study reported that *AdipoR1* knockout (KO) mice did not develop insulin resistance while *AdipoR2* KO mice were actually protected from developing this pathology when fed a high-fat diet^4^. In addition to this discrepancy, it has recently been shown that the *AdipoR1* KO developed retinal degeneration while the KO of adiponectin did not^5^, suggesting that ADIPOR1 can definitively act independently of adiponectin. This function of ADIPOR1 in the eye rather than in glucose metabolism is further underscored by the novel discovery of two different *ADIPOR1* mutations that cause retinitis pigmentosa in humans^6,7^ while mutations causing insulin resistance have thus far not been identified^8^.

In the previous study of *AdipoR1* KO-induced retinal degeneration it was shown that the KO mice had developed a flecked retina, an accumulation of subretinal macrophages/microglia, highly diminished electroretinograms (ERGs) prior to significant photoreceptor loss, and a severe preferential deficiency of docosahexaenoic acid (DHA) in the eye^5^. In an effort to better understand this biology, we have identified a specific anti-ADIPOR1 antibody capable of discriminating between wildtype (WT) and KO cells and tissues. We profiled ADIPOR1 protein distribution across mouse tissues and found that unlike its near-ubiquitous mRNA levels^1^ this protein is enriched specifically in the eye and the brain with little relative expression in the other tissues, such as liver and skeletal muscle. Furthermore, in the retina we observed ADIPOR1 expression in photoreceptors and the retinal pigment epithelium (RPE). We then went on to demonstrate a role for ADIPOR1 post-development by knocking it out from adult floxed animals using either RPE or photoreceptor-specific *Cre* expression which resulted in decreased expression of multiple retinal markers, establishing an important role for this protein in both of these cell types.

Additionally, we characterized protein levels in *AdipoR1* KO mouse retinas to show that these mice are depleted of RHODOPSIN and other visual system proteins by three weeks of age, presenting an alternative hypothesis for the observed low DHA levels. We also profiled the membrane frizzled related protein mutant mouse^9^ (a de facto KO model known as *Mfrp*^*rd6*^) that exhibits a retinal phenotype very similar to the *AdipoR1* KO mice. Remarkably, absence of MFRP caused a loss of ADIPOR1 specifically in the RPE layer, while expression of ADIPOR1 persisted in the photoreceptors. Lastly, we profiled gene expression between *AdipoR1* WT, heterozygous (HET), and KO animals and found that the interphotoreceptor retinoid-binding protein (IRBP, aka RBP3) was strongly upregulated in KO eyes prior to retinal degeneration, suggesting retinoid metabolism dysfunction. Subsequent analysis of *Mfrp*^*rd6*^ mouse eyes also identified an IRBP increase in these mice prior to retinal degeneration akin to the *AdipoR1* KO mice.

## Results

### Antibody screen identifies a specific anti-ADIPOR1 antibody that highlights restricted protein expression among different tissues

**Figure 1:**
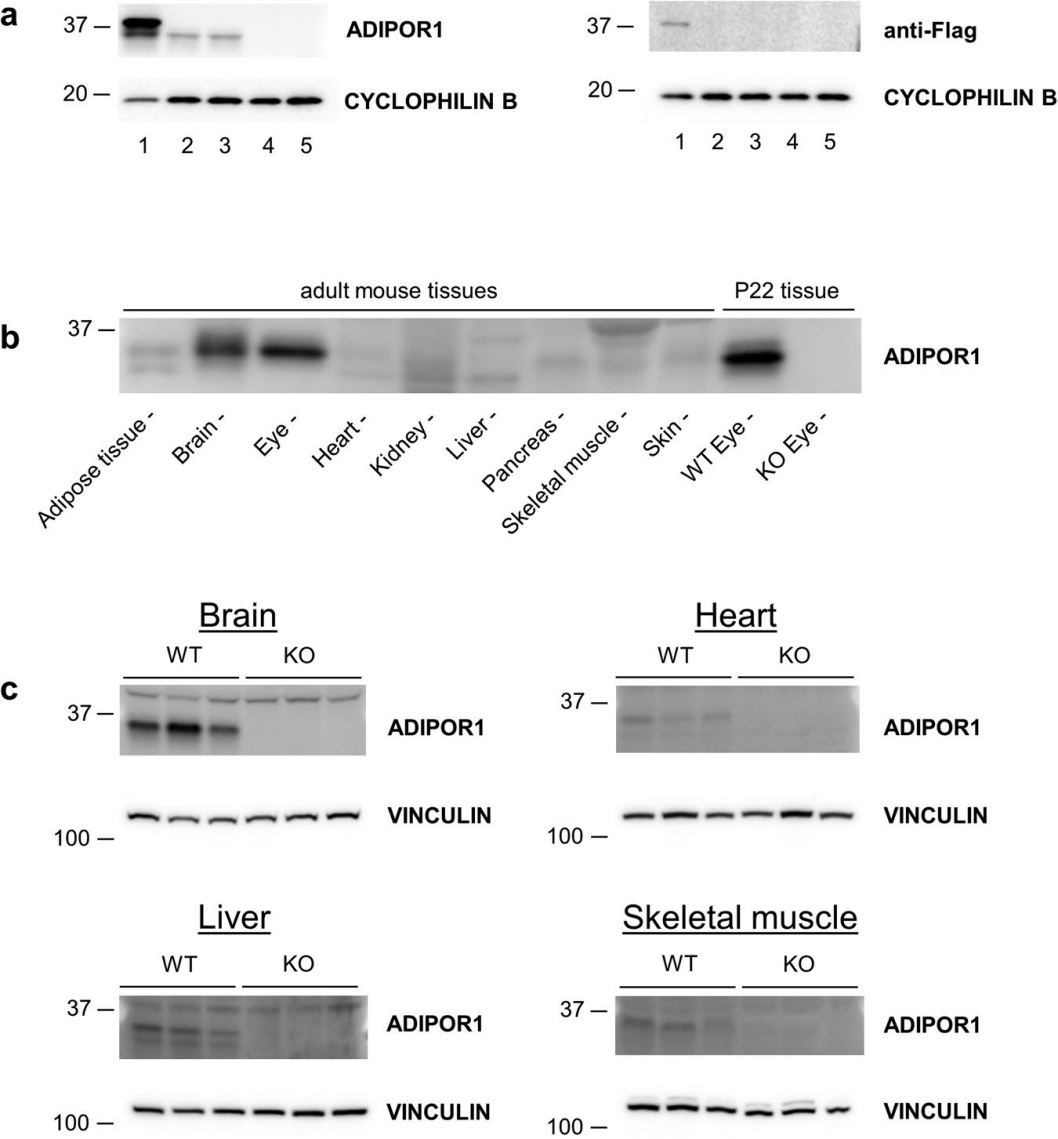
ADIPOR1 protein expression in mouse tissues. (**a**) Anti-ADIPOR1 antibody (IBL) can recognize endogenous and exogenous ADIPOR1. Lanes: 1 – Flag-ADIPOR1 transfected HEK293T, 2 – WT/Untransfected HEK293T, 3 – CRISPR negative control transfected HEK293T, 4 – *ADIPOR1* KO HEK293T clone 4, *ADIPOR1* KO HEK293T clone 5. Anti-Flag antibody (CST) was used to detect exogenous tagged ADIPOR1. CYCLOPHILIN B was used as a loading control. (**b**) ADIPOR1 protein expression profile from different tissues. Adult mouse tissues and P22 mouse eye tissue were profiled. ADIPOR1 is enriched in the eye and brain. The antibody retains specificity in tissue samples as based on WT and KO eye tissue discrimination. (**c**) ADIPOR1 protein is present in non-nervous tissues. ADIPOR1 protein signal can be appreciated in liver, heart, and skeletal muscle when ran without brain or eye samples, confirming protein presence in those tissues. Four and a half month old *AdipoR1* WT and KO mice were used for tissue collection. Each lane represents a sample from an individual mouse. VINCULIN was used as a loading control. For (**a**) and (**c**) each membrane was cut and probed separately for the analyzed protein and the loading control.

In order to gain further insights into ADIPOR1 biology, we first wanted to identify an antibody to study ADIPOR1 protein levels as well as cell and tissue distribution. While a number of publications on ADIPOR1 have relied on transcript levels to profile its expression^1,5,10^, perhaps in part due to lacking a trustworthy antibody reagent, this dependency on transcript levels may be misleading since mRNA levels do not always correlate well with protein^11,12^, especially across different tissues^13^. Therefore, we screened a number of commercially available antibodies for their ability to detect ADIPOR1. We utilized HEK293T cells as a model to profile the antibodies as these cells are easy to transfect and have been reported to express endogenous ADIPOR1 protein^14^. First, we tested whether we could detect exogenous Flag-tagged ADIPOR1 from a plasmid transfection. During sample optimization for western blot analysis, we noted that this overexpressed ADIPOR1 exhibited temperature sensitivity as heating the protein samples to a temperature of 60 °C or greater led to a loss of detectable anti-Flag signal (Supplementary Fig. 1). Therefore, we chose to only heat protein samples to a temperature of 37 °C for subsequent SDS-PAGE analysis. To generate a negative control of antibody specificity for our study, we utilized CRISPR-Cas9 to knock out *ADIPOR1* from HEK293T cells and confirmed two independent clones to be *ADIPOR1* KOs by DNA sequencing. Then we tested fifteen anti-ADIPOR1 antibodies on *ADIPOR1*-plasmid transfected, WT, CRISPR control (treated with non-genome targeting gRNAs), and *ADIPOR1* KO HEK293T cells. This antibody screen revealed that only five of these reagents could detect exogenous ADIPOR1 while only one antibody (IBL - ADIPOR1) could detect the endogenous protein as determined by the antibody’s ability to detect a protein of the predicted molecular weight in the WT but not KO samples (Fig. 1a, Supplementary Fig. 2, Table S1). Using this identified antibody, we then re-tested the heat sensitivity of the endogenous ADIPOR1 protein and found that like its transfected version and similar to other membrane proteins^15,16^, it was also highly vulnerable to heat as even 1 minute at 95 °C strongly reduced the detected signal (Supplementary Fig. 1).

Next, we wanted to test this antibody for its ability to detect endogenous ADIPOR1 from tissue samples as well as to look at ADIPOR1 protein distribution. A western blot of different mouse tissues, including a WT and KO mouse eye control, revealed successful antibody detection of the endogenous ADIPOR1 protein as well as a strong signal in the eye and the brain, with only faint bands of the predicted weight found in the other analyzed tissues (Fig. 1b, Supplementary Fig. 3). To further determine if these very low abundance bands were real ADIPOR1 and/or if ADIPOR1 ran at a different molecular weight in non-central nervous system tissues, as the strongest bands in these sample lanes were not found in the predicted molecular weight range, we ran WT and KO samples of brain, liver, muscle, and heart tissues on separate blots. This additional analysis confirmed that ADIPOR1 is indeed present in the liver, muscle, and heart, but at much lower levels (Fig. 1c, Supplementary Fig. 3).

### ADIPOR1 protein is found in the human and mouse neural retina and RPE

**Figure 2:**
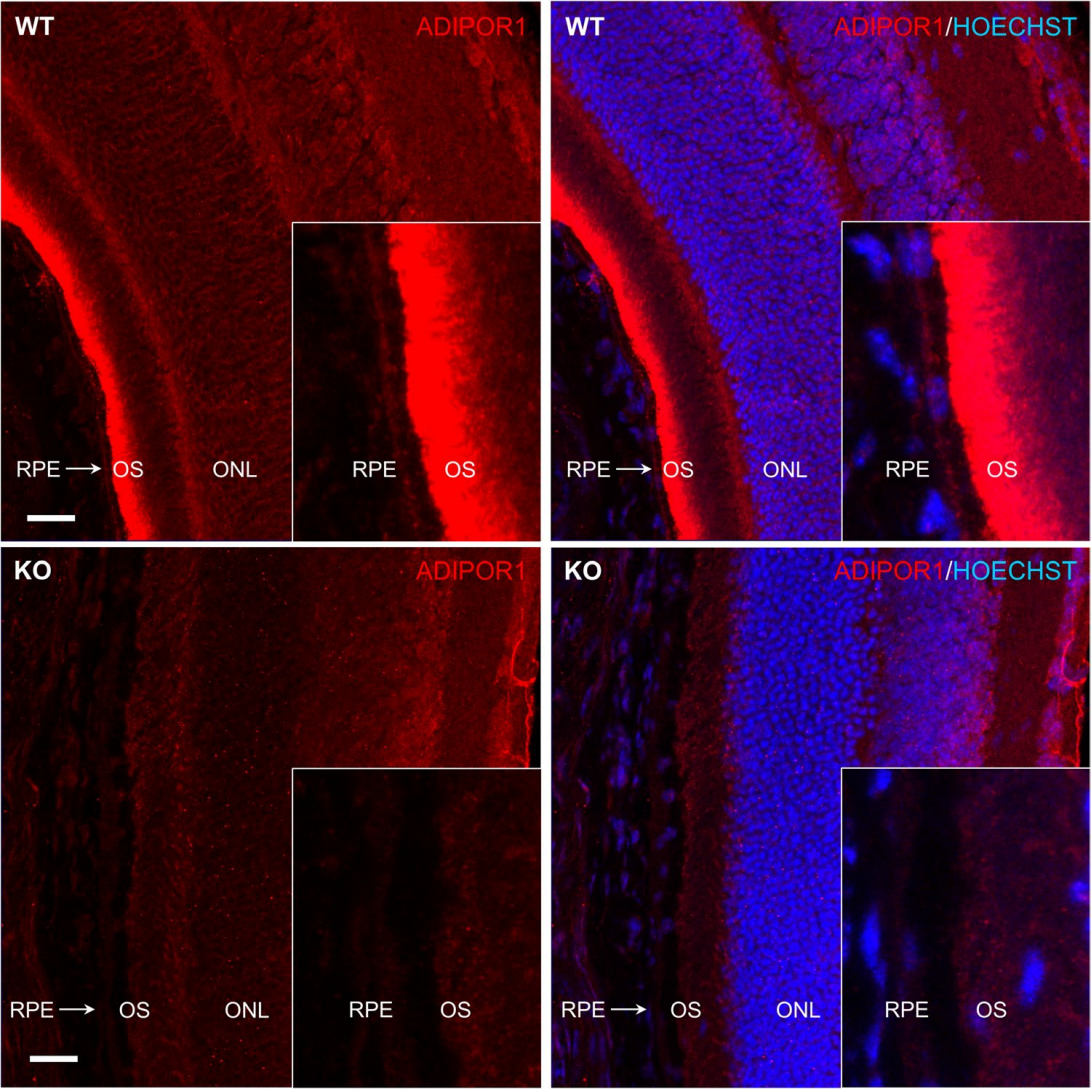
ADIPOR1 is expressed in the neural retina and RPE. IHC for ADIPOR1 protein expression. P21 mouse eyes of *AdipoR1* WT or KO genotypes were stained with the anti-ADIPOR1 antibody (IBL). ADIPOR1 signal is observed in the neural retina and the RPE with strongest expression in the OS. Scale bar = 20 µm. Z-stacks were acquired and the maximum intensity projection is displayed.

Following our western blot profile, we analyzed the expression of ADIPOR1 in the retina by immunohistochemistry (IHC) using frozen cryosections of three week old WT and KO mice. This analysis confirmed that the IBL antibody was able to detect ADIPOR1 with IHC and it highlighted that this protein is present in the retina with the strongest signal appearing in the photoreceptor outer segments (OS). Weaker signal intensity appeared in the rest of the neural retina including the outer nuclear layer (ONL), and unlike a previous report^7^, the RPE (Fig. 2). Following the determination of ADIPOR1 expression in the mouse retina, we wanted to compare this profile with human expression. First, we performed *in situ* hybridization (ISH) for mRNA levels of *ADIPOR1* in the human retina using RNAscope (Supplementary Fig. 4). This analysis showed *ADIPOR1* mRNA presence throughout the neural retina and the RPE. We then performed IHC on paraffin-embedded sections of human retinas to test for protein expression of ADIPOR1 and observed a surprising enrichment of ADIPOR1 only on the apical side of the RPE layer (Supplementary Fig. 4). To confirm whether ADIPOR1 protein was truly absent from the neural retina or whether the antibody was less adapt at detecting paraffin embedded epitopes, we performed western blotting on separately dissected human eye samples of neural retina and posterior eye cup that contains the RPE layer. Blotting for RPE65 and RHODOPSIN, as markers of the RPE and the photoreceptors/neural retina, respectively, we confirmed that the retinal layers were indeed separated in these samples and ADIPOR1 protein was found in both layers (Supplementary Fig. 4).

### *AdipoR1* KO mice are largely normal at two weeks but have a compromised visual system by three weeks of age

**Figure 3:**
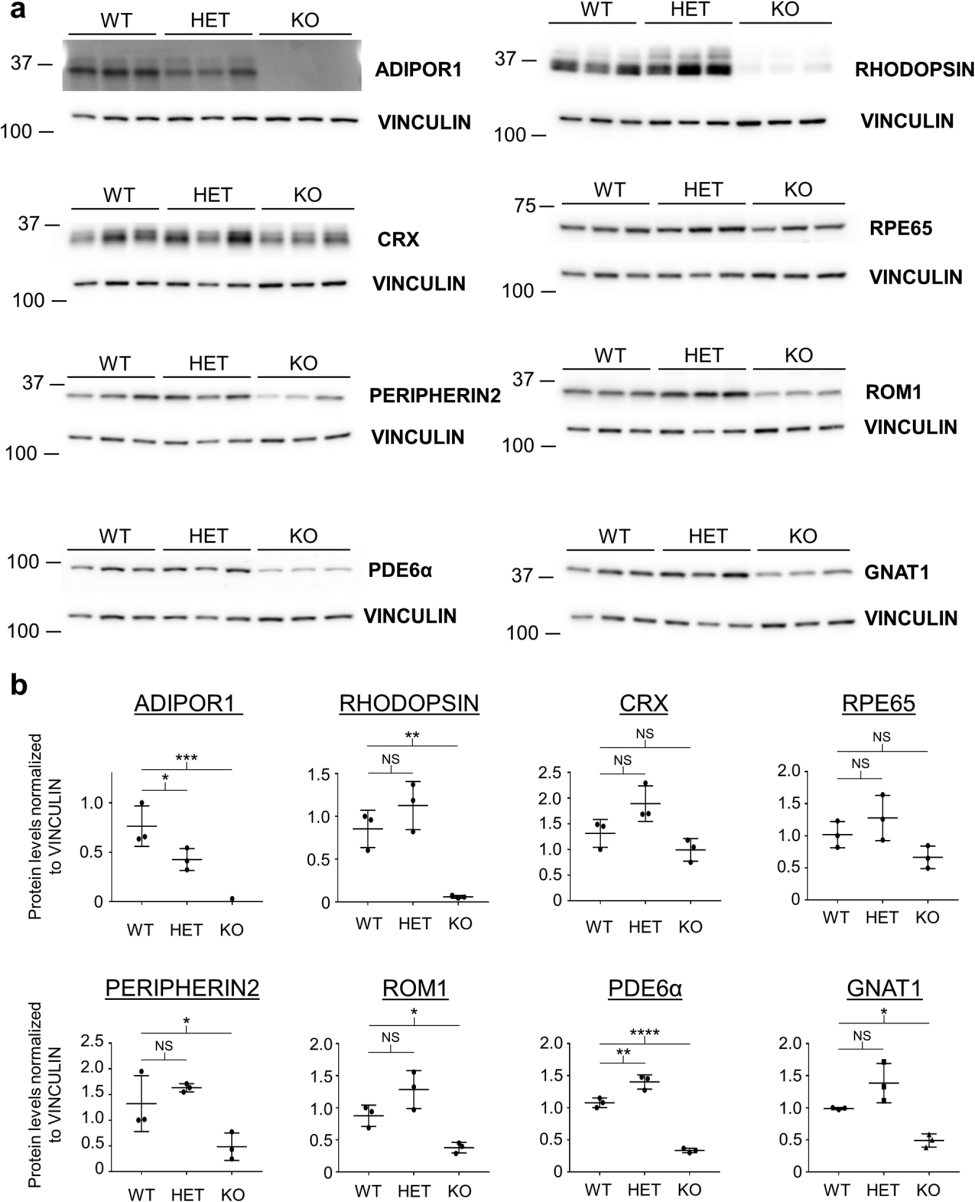
Three week old *AdipoR1* KO mice exhibit deficient expression of visual system proteins. (**a**) Western blots of P22 *AdipoR1* WT, HET, and KO mouse eyes are shown. ADIPOR1 as well as known visual system proteins were profiled. VINCULIN was used as a loading control. Each lane represents a sample from an individual mouse, n = 3. KO mice exhibit reduced levels of RHODOPSIN, PERIPHERIN-2, ROM1, PDE6α, and GNAT1. Each membrane was cut and probed separately for the analyzed protein and the loading control. (**b**) Densitometry quantification of western blots in (a). ANOVA (α = 0.05) with Dunnett’s multiple comparisons test (WT set as control) was used. Error bars represent standard deviation. *p < 0.05, **p < 0.01, ***p < 0.001, ****p < 0.0001, NS = not significant. P values: ADIPOR1, WT vs HET p = 0.0409, WT vs KO p = 0.0008; CRX = WT vs HET p = 0.0811, WT vs KO p = 0.3451; PDE6α, WT vs HET p = 0.0045, WT vs KO p = 0.0001; RHODOPSIN = WT vs HET p = 0.2541, WT vs KO p = 0.0058; GNAT1 = WT vs HET p = 0.0706, WT vs KO p = 0.0298; RPE65 = WT vs HET p = 0.4107, WT vs KO p = 0.2346; PERIPHERIN2 = WT vs HET p = 0.4984, WT vs KO p = 0.0473; ROM1 = WT vs HET p = 0.0825, WT vs KO p = 0.0409.

Since the previous *AdipoR1* KO study had shown subtle retinal degeneration but profound loss of ERGs in 3–4 week old animals, we wondered how the expression of visual system proteins was affected at this early stage prior to pronounced photoreceptor loss^5^. First, we looked at RHODOPSIN levels in *AdipoR1* KO eyes at postnatal day 22 (P22) by western blot. This analysis highlighted a surprisingly sharp loss of RHODOPSIN at this early age (Fig. 3a,b) in the KO but not the HET or WT samples. Then we profiled the eyes for levels of CRX, a photoreceptor transcription factor whose levels would be indicative of ONL thinning rather than OS shortening. The levels of CRX were statistically normal, however, supporting the prior data that the ONL is still largely intact at this age^5^. Next, we further profiled two OS-associated structural proteins, PERIPHERIN-2 and ROM1, and found the levels of both to be significantly decreased in the KO eyes, suggesting that the OS in three week old *AdipoR1* KO animals are compromised. Additionally, we looked at the levels of three proteins involved in vision, RPE65, PDE6α, and GNAT1, and detected a significant drop in levels of PDE6α and GNAT1 and a downward trend in RPE65, demonstrating that the rest of the visual cascade is also negatively compromised in the KO animals.

**Figure 4:**
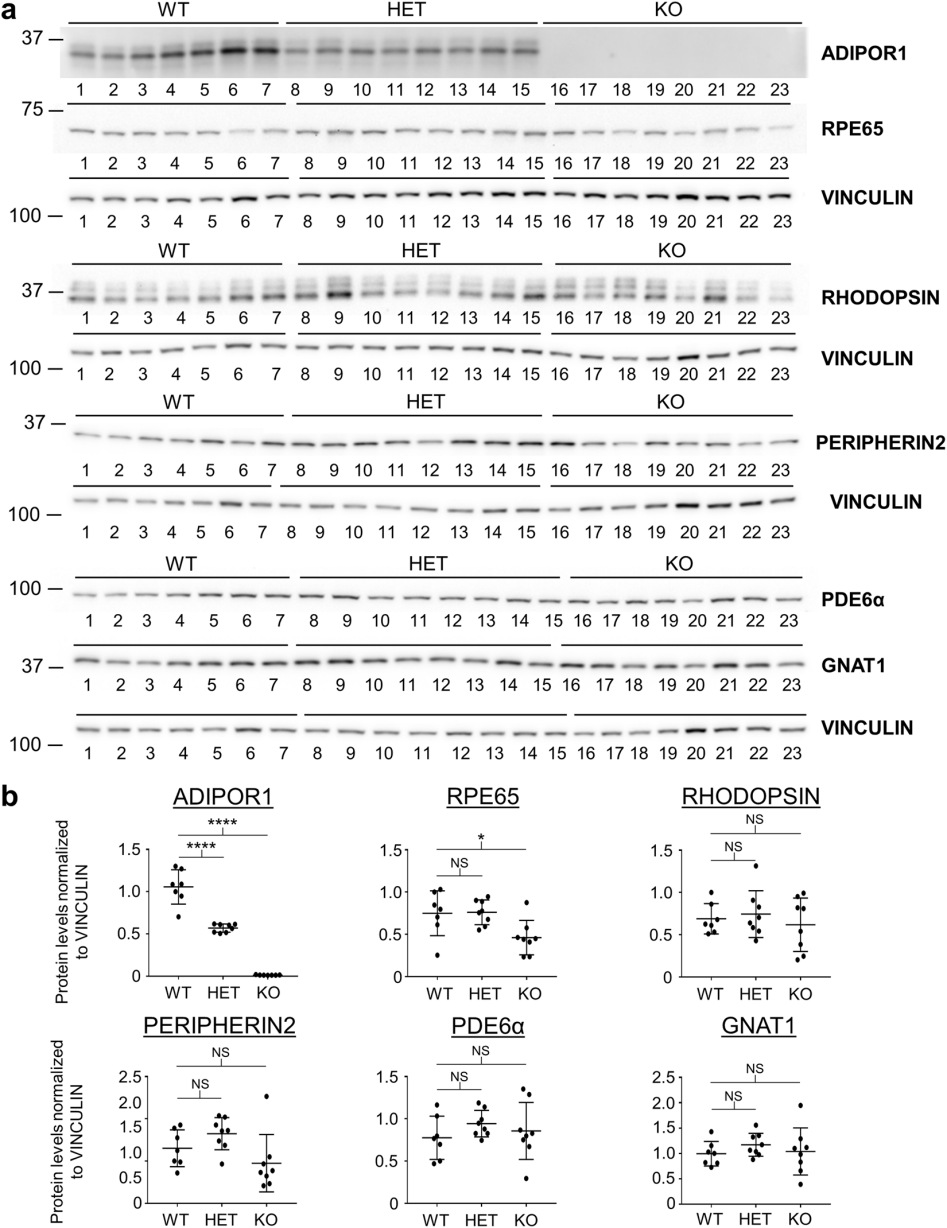
Two week old *AdipoR1* KO mice appear normal. (**a**) Western blots of P15 *AdipoR1* WT, HET, and KO mouse eyes are shown. ADIPOR1 as well as known visual system proteins were profiled. VINCULIN was used as a loading control. Each lane represents a sample from an individual mouse, n = 7 for WT, n = 8 for HET, n = 8 for KO. KO mice appear normal except for a possible decrease of RPE65. Each membrane was cut and probed separately for the analyzed proteins and the loading control. (**b**) Densitometry quantification of western blots in (a). ANOVA (α = 0.05) with Dunnett’s multiple comparisons test (WT set as control) was used. Error bars represent standard deviation. *p < 0.05, **p < 0.01, ***p < 0.001, ****p < 0.0001, NS = not significant. P values: ADIPOR1, WT vs HET p = 0.0001, WT vs KO p = 0.0001; RPE65, WT vs HET p = 0.9917, WT vs KO p = 0.0261; RHODOPSIN, WT vs HET p = 0.8916, WT vs KO p = 0.8314; PERIPHERIN2, WT vs HET p = 0.3510, WT vs KO p = 0.3317; PDE6α, WT vs HET p = 0.3728, WT vs KO p = 0.7756; GNAT1, WT vs HET p = 0.5035, WT vs KO p = 0.9541.

We wondered if the visual system was disturbed even earlier and profiled most of the proteins from our P22 profile again using P15 mouse eyes. With the exception of a small decrease in RPE65, none of the other profiled proteins reached a statistically significant decrease at this younger age (Fig. 4a,b), suggesting that retinal degeneration begins later than P15 in these animals. Additionally, apart from a slight increase of PDE6α in the P22 HETs, the protein levels of both WT and HET samples at P15 and P22 showed mostly equal levels, indicating that half of the normal ADIPOR1 expression is sufficient for retinal stability.

### ADIPOR1 loss in photoreceptors or RPE of adult animals decreases expression of markers critical for vision

After determining that ADIPOR1 is necessary for the visual system since its absence during development results in retinal degeneration and early-onset blindness, we wondered if ADIPOR1 is also needed post-development in adult animals and whether it is needed in both photoreceptors and the RPE. We generated AAV-*Cre* viruses where *Cre* is driven by a (1) constitutive *CMV* promoter, (2) *VMD2* (aka *BEST1*), an RPE specific promoter, or (3) *IRBP*, a photoreceptor specific promoter. Then we injected these AAVs subretinally into the eyes of nine month old WT or floxed *AdipoR1* mice that would lose their ADIPOR1 protein expression upon Cre-induced recombination.

To assess any retinal damage stemming from AAV-*Cre* induced ADIPOR1 loss, we measured retinal thickness close to the site of virus injection using optical coherence tomography (OCT) at one and three months post injection (Supplementary Fig. 5). At both time points, no statistically significant difference was reached for retinal thinning between WT and floxed injected animals, a comparison that would be indicative of retinal thinning arising due to ADIPOR1 loss alone.

However, retinal thinning was observed in the injected animals when compared to naïve (uninjected) animals, with the largest decrease observed in the *CMV-Cre* and *VMD2-Cre* treated floxed groups at three months post injection. Yet, this decrease occurred in both WT and floxed animals, likely due to Cre toxicity^17,18^, thus we could not verify the role of ADIPOR1 loss based on OCT data alone. Since our OCT analysis only evaluated a relatively minor region of the retina near the injection site, where Cre overexpression-induced death would likely be strongest, we decided to analyze the whole mouse eyes to look for effects of the KO on a larger area. To determine whole eye effects we analyzed retinal cell marker protein expression in the injected mice at 5 months post injection by western blot.

**Figure 5:**
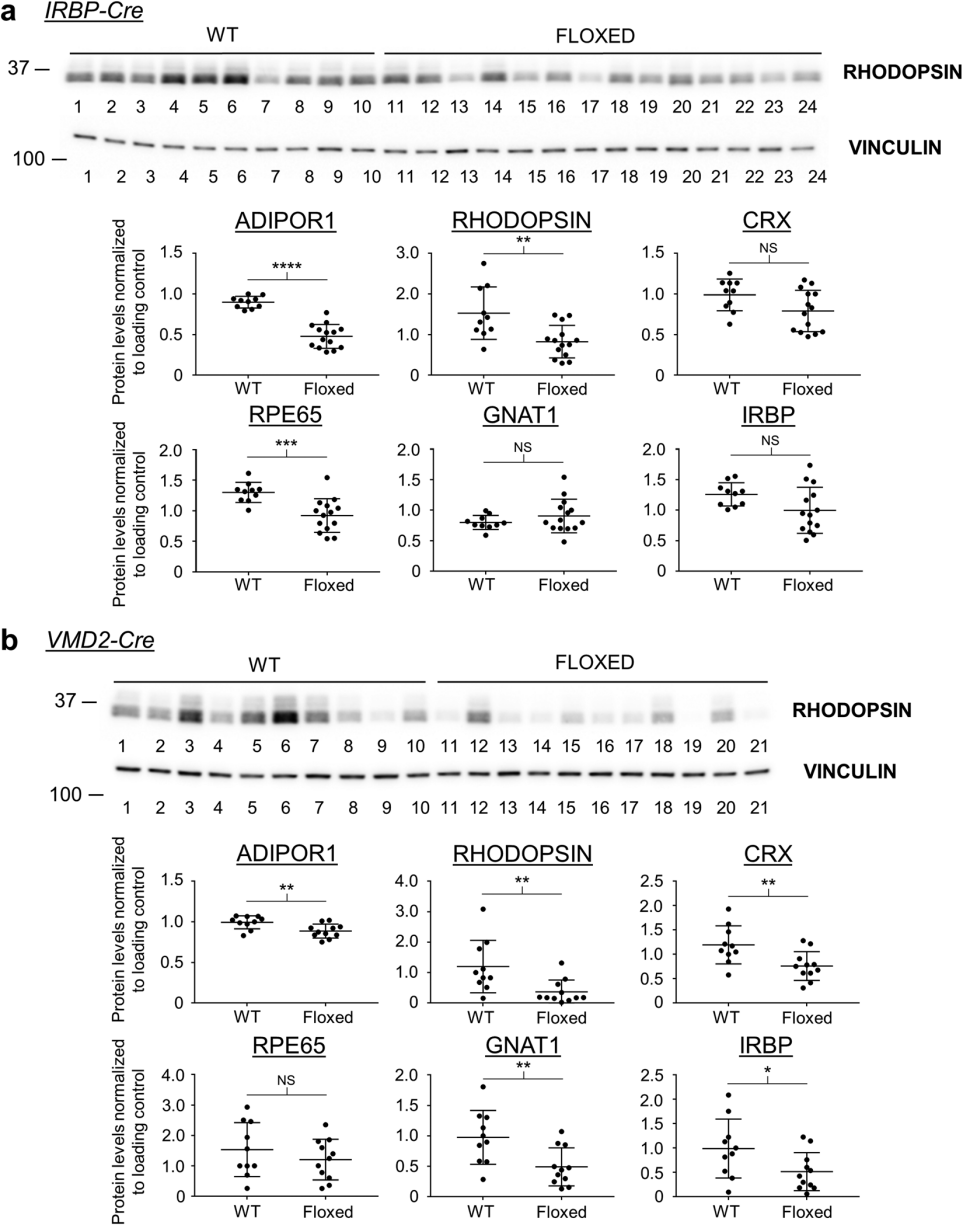
AAV-*Cre* treated adult floxed mice show decreased expression of markers critical for vision. (**a**) Western blot for RHODOPSIN of *IRBP-Cre* treated mouse eyes is shown. VINCULIN or α-TUBULIN was used as a loading control. Each lane represents an individual eye from a mouse of that genotype, n = 10 for WT, n = 14 for Floxed. Densitometry quantification for RHODOPSIN and additional western blots (see Supplementary Fig. 8) is shown below. *IRBP-Cre* treated mice show a large drop in ADIPOR1, confirming Cre induced KO of this protein. These mice also display a significant drop of RHODOPSIN and RPE65. *IRBP* group - P values: ADIPOR1 - p = < 0.0001; RHODOPSIN - p = 0.0033; CRX - p = 0.0512; RPE65 - p = 0.0009; GNAT1 - p = 0.2680; IRBP - p = 0.0570. (**b**) Western blot for RHODOPSIN of *VMD2-Cre* treated mouse eyes is shown. VINCULIN or α-TUBULIN was used as a loading control. Each lane represents an individual eye from a mouse of that genotype, n = 10 for WT, n = 11 for Floxed. Densitometry quantification for RHODOPSIN and additional western blots (see Supplementary Fig. 9) is shown below. *VMD2-Cre* treated mice show a small drop in ADIPOR1 consistent with a KO of this protein only in the RPE layer. These mice also display a significant drop of RHODOPSIN, CRX, GNAT1, and IRBP. *VMD2* group - P values: ADIPOR1 - p = 0.0080; RHODOPSIN - p = 0.0092; CRX - p = 0.0093; RPE65 - p = 0.3471; GNAT1 - p = 0.0087; IRBP - p = 0.0442. Unpaired two tailed t-test was used. *p < 0.05, **p < 0.01, ***p < 0.001, ****p < 0.0001, NS = not significant. Error bars represent standard deviation. Mice were taken down at 5 months post AAV injection. For western blots in (**a**,**b**) each membrane was cut and probed separately for the analyzed protein and the loading control.

First, we tested the levels of RHODOPSIN and CRX in whole eyes from naïve control age-matched litters and observed equal RHODOPSIN and CRX levels between WT and floxed animals with little sample-to-sample variability (Supplementary Fig. 6). Then we tested the *CMV-Cre*, *IRBP-Cre*, and *VMD2-Cre* treated animals for ADIPOR1, RHODOPSIN, CRX, RPE65, GNAT1, and IRBP (Fig. 5a,b, Supplementary Figs 7, 8 and 9). Unlike the naïve control mice, western blots of the AAV-*Cre* treated mice showed much more variable RHODOPSIN and CRX levels. For the *CMV-Cre* group, we observed a 37% decrease for ADIPOR1 in the floxed animals compared to WT controls supporting Cre-induced KO (Supplementary Fig. 7). However, despite a downward trend for floxed vs WT samples, blots for RHODOPSIN, CRX, GNAT1, and IRBP did not show a statistically significant difference between the two genotypes. Nevertheless, the levels of RPE65 were significantly decreased in the floxed mice as compared to WT controls. The western blot analysis of the *IRBP-Cre* and *VMD2-Cre* treated animals showed more statistically significant differences that further supported the hypothesis that *AdipoR1* KO leads to a decrease in expression of markers critical for vision in adult animals.

For the *IRBP-Cre* treated group, which appeared less variable than the *CMV-Cre* group reviewed above, western blot analysis showed a 47% decrease in ADIPOR1 for the floxed animals vs WT controls (Fig. 5a, Supplementary Fig. 8). The levels of RHODOPSIN and RPE65 were also statistically significantly decreased in the floxed mice. The levels of CRX, GNAT1, and IRBP were not significantly decreased though, suggesting that despite a loss of RHODOPSIN, the photoreceptors had not yet succumbed to cell death which is consistent with the OCT data showing no significant retinal thinning of *IRBP-Cre* treated vs naïve mice. In the *VMD2-Cre* treated group, we detected the smallest decrease of ADIPOR1 for the floxed vs WT animals, only 11%, consistent with the cell-type specific KO of *AdipoR1* in the RPE cells alone (Fig. 5b, Supplementary Fig. 9). However, despite the smaller loss of ADIPOR1, *VMD2-Cre* treated floxed animals demonstrated a statistically significant decrease in five of the six proteins we analyzed: ADIPOR1, RHODOPSIN, CRX, GNAT1, and IRBP.

### ADIPOR1 RPE expression is diminished in the *Mfrp*^*rd6*^ mouse

**Figure 6:**
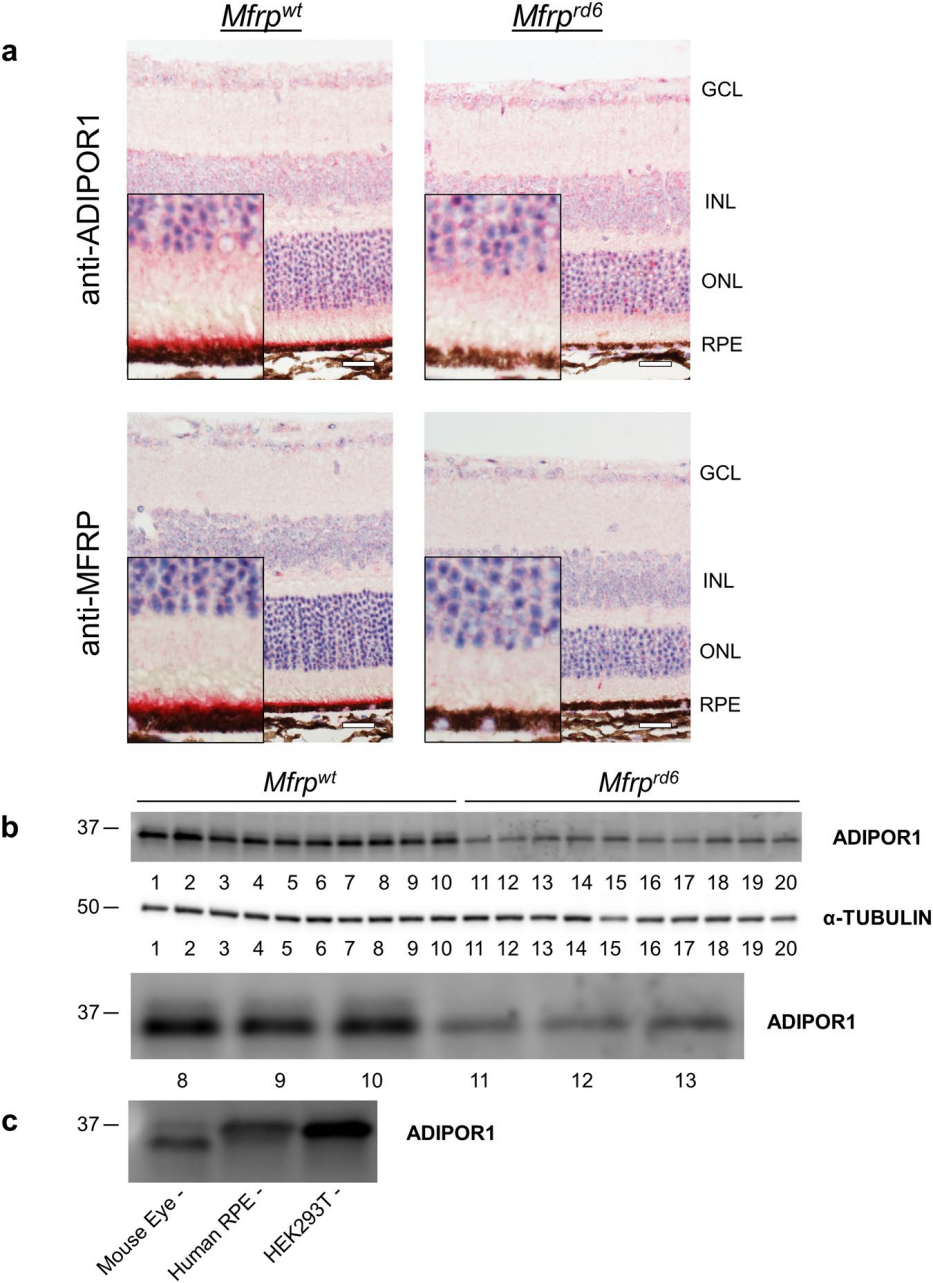
*Mfrp*^*rd6*^ mice lack ADIPOR1 in their RPE layer. (**a**) IHC on P32 *Mfrp*^*wt*^ and *Mfrp*^*rd6*^ mouse eyes. Anti-ADIPOR1 staining was performed on *Mfrp*^*wt*^ controls and *Mfrp*^*rd6*^ mouse eyes. Anti-MFRP staining below confirms genotypes. Inset shows enlarged image near RPE layer. ADIPOR1 signal is lost in the RPE of *Mfrp*^*rd6*^ animals. ADIPOR1/MFRP is observed via the red reaction product. Scale bar = 20 µm. (**b**) Western blot for ADIPOR1 using *Mfrp*^*wt*^ and *Mfrp*^*rd6*^ mouse eyes of age P21. α-TUBULIN was used as a loading control. Each lane represents an eye from a mouse of that genotype and eyes were loaded in pairs from individual animals (5 *Mfrp*^*wt*^ and 5 *Mfrp*^*rd6*^ individual animals), n = 10 eyes for both genotypes. The membrane was cut and probed separately for the analyzed protein and the loading control. Below, a zoomed in image of lanes 8–13 highlights the absence of the top ADIPOR1 band from the *Mfrp*^*rd6*^ animals. (**c**) Western blot for ADIPOR1 comparing samples from a mouse eye, human stem cell derived RPE, and HEK293T cells. RPE and HEK293T ADIPOR1 runs at the same molecular weight as the top band from the mouse eye ADIPOR1 doublet. RPE cells were derived from H9 hESCs and matured for three months before analysis.

The molecular function of ADIPOR1 in the retina is currently unclear. To identify potential pathways affected by ADIPOR1, we examined the *Mfrp*^*rd6*^ mouse^9^. Retinal phenotypes in the *Mfrp*^*rd6*^ mice are similar to those observed in the *AdipoR1* KO. Both develop a flecked retina fundus appearance, accumulate subretinal macrophages, exhibit ONL thinning starting at approximately 1 month of age, and show diminished ERGs before significant photoreceptor loss^9,19,20^. Additionally, like ADIPOR1, MFRP has been reported to be expressed on the apical side of the RPE layer in mice^9^ and we have observed this same expression pattern in the human retina (Supplementary Fig. 4). Due to these similarities, we wondered if ADIPOR1 expression was perturbed in the absence of MFRP or vice versa. First, we performed IHC on *AdipoR1* WT, HET, and KO mouse retinas for MFRP and detected equally enriched apical RPE expression for all genotypes (Supplementary Fig. 10). Then, we performed IHC for ADIPOR1 on *Mfrp*^*rd6*^ retinas and observed a striking loss of ADIPOR1 signal specifically in the RPE layer (Fig. 6a). Western blotting for ADIPOR1 expression in *Mfrp*^*rd6*^ mouse eyes showed that at P21, an age with little to no ONL thinning^20^, ADIPOR1 is dramatically decreased (Fig. 6b). We also took greater notice of the ADIPOR1 doublet running pattern on a western blot. Notably, the top fainter band of ADIPOR1 appeared to be missing in the *Mfrp*^*rd6*^ mouse eyes. We probed this running pattern further by comparing a whole mouse eye, human stem cell-derived RPE cells, and HEK293T cell lysate side by side and saw that the RPE and HEK293T ADIPOR1 tends to run higher than the darker mouse eye band (Fig. 6c), suggesting that the missing top band in *Mfrp*^*rd6*^ eyes represents the loss of ADIPOR1 in their RPE layer.

### RNA-seq profile of *AdipoR1* KO model mice identifies an increase in *Irbp* levels at P15

**Figure 7:**
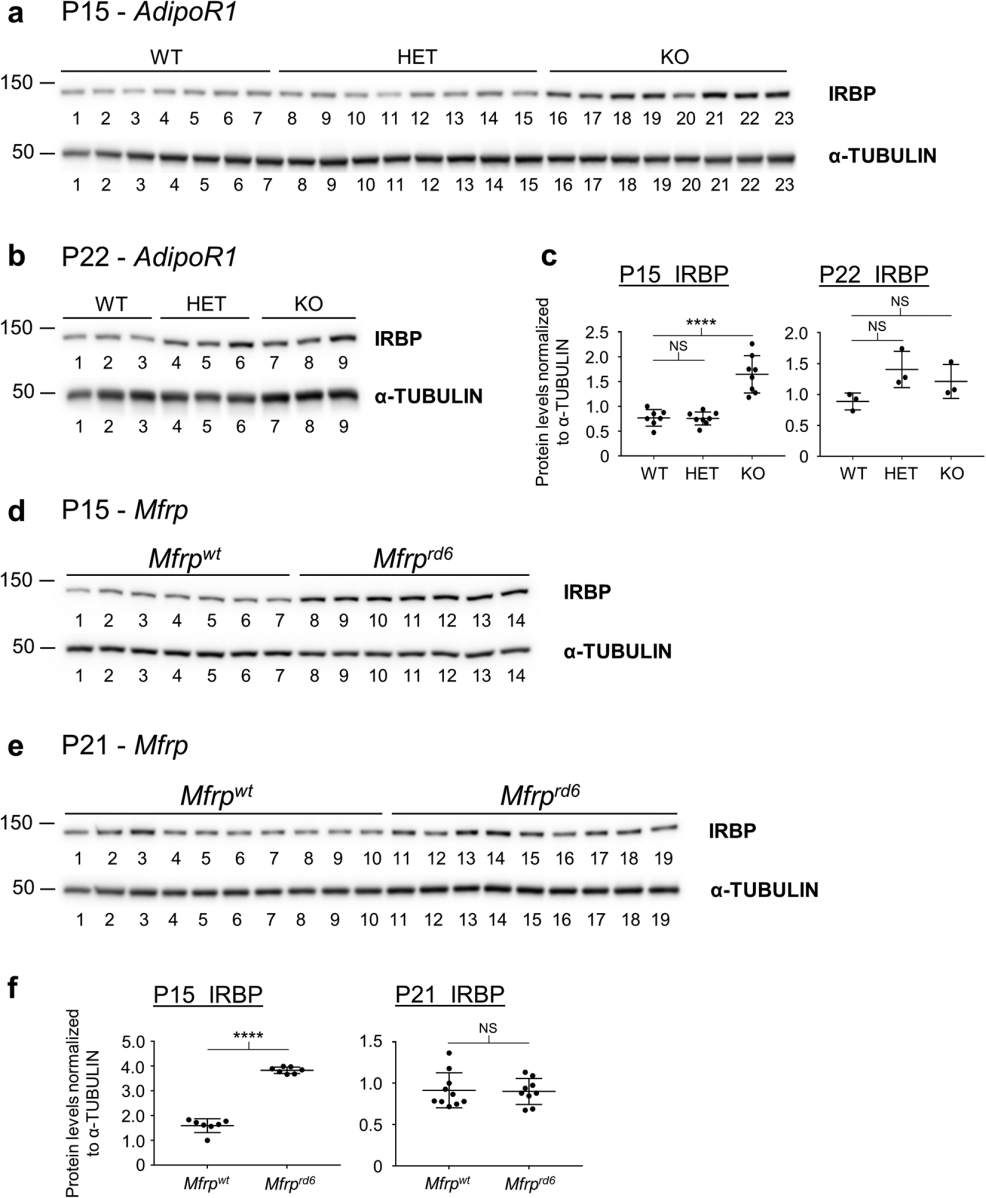
*AdipoR1* KO mice and *Mfrp*^*rd6*^ mice have an upregulation of IRBP at 2 weeks of age. (**a**) Western blot of P15 *AdipoR1* WT, HET, and KO mouse eyes is shown. α-TUBULIN was used as a loading control. Each lane represents a sample from an individual mouse, n = 7 for WT, n = 8 for HET, n = 8 for KO. (**b**) Western blot of P22 *AdipoR1* WT, HET, and KO mouse eyes is shown. α-TUBULIN was used as a loading control. Each lane represents a sample from an individual mouse, n = 3. (**c**) Densitometry quantification of blots from (**a**,**b**). (**d**) Western blot of P15 *Mfrp*^*wt*^ or *Mfrp*^*rd6*^ mouse eyes is shown. α-TUBULIN was used as a loading control. Each lane represents a sample from an individual mouse, n = 7 for *Mfrp*^*wt*^, n = 7 for *Mfrp*^*rd6*^. (**e**) Western blot of P21 *Mfrp*^*wt*^ or *Mfrp*^*rd6*^ mouse eyes is shown. α-TUBULIN was used as a loading control. Each lane represents an eye from a mouse of that genotype and eyes were loaded as pairs from individual animals (5 *Mfrp*^*wt*^ and 5 *Mfrp*^*rd6*^ individual animals), n = 10 eyes for *Mfrp*^*wt*^ and n = 9 for *Mfrp*^*rd6*^ (one eye was excluded due to low protein levels). (**f**) Densitometry analysis of blots from (**d**,**e**). *p < 0.05, **p < 0.01, ***p < 0.001, ****p < 0.0001, NS = not significant. ANOVA (α = 0.05) with Dunnett’s multiple comparisons test (WT set as control) was used in (**c**). Unpaired two tailed t-test was used in (**f**). Error bars represent standard deviation. P values: for part (**c**). IRBP P15 *AdipoR1*: WT vs HET - p = 0.9940, WT vs KO - p = 0.0001. IRBP P22 *AdipoR1*: WT vs HET - p = 0.0714, WT vs KO - p = 0.2560. For part (**f**). IRBP P15 *Mfrp*^*rd6*^: p < 0.0001, IRBP P21 *Mfrp*^*rd6*^ p = 0.8777. For all western blots, each membrane was cut and probed separately for the analyzed protein and the loading control.

**Figure 8:**
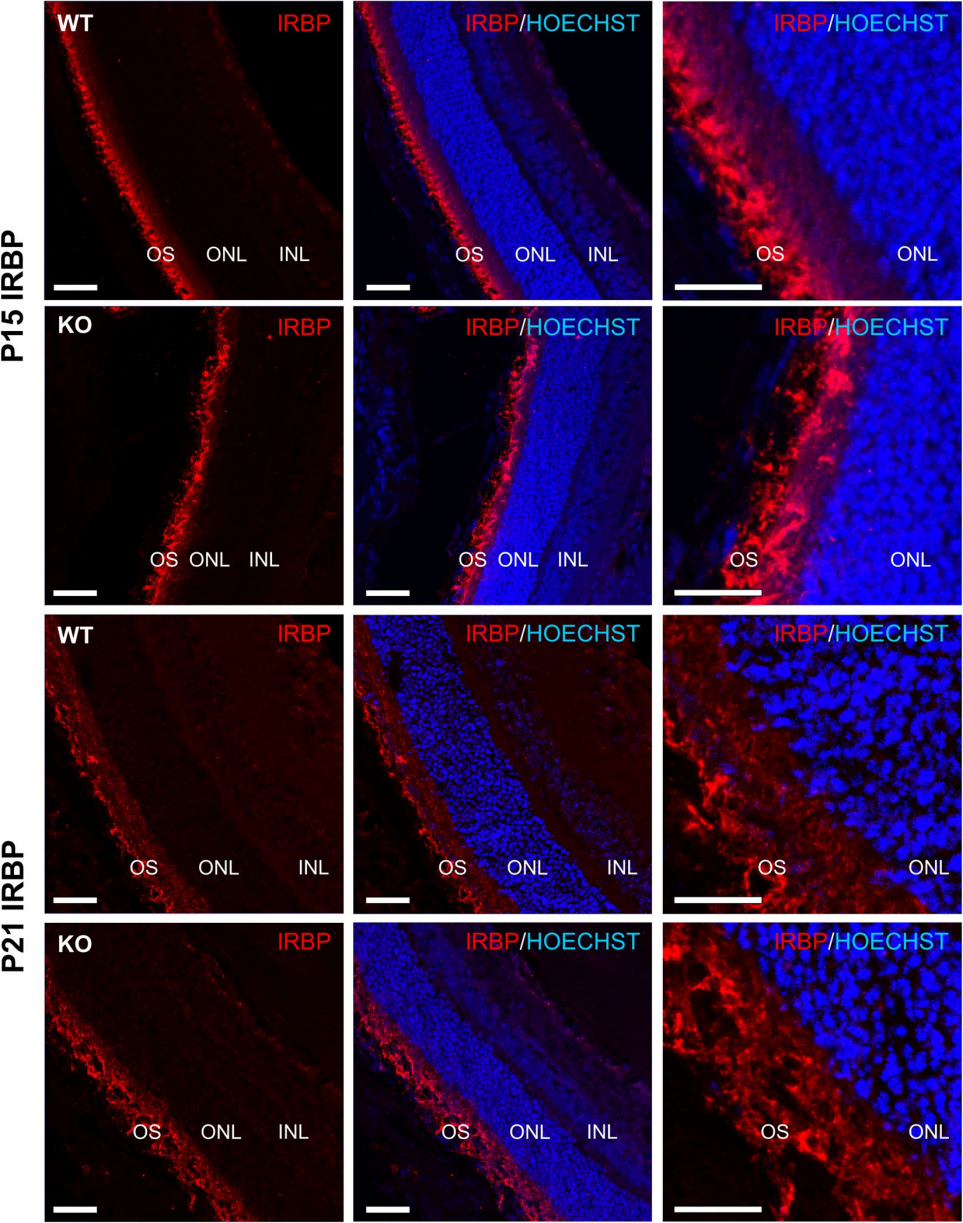
IRBP is mislocalized in the *AdipoR1* KO mice. IHC of P15 and P21 mouse eyes of *AdipoR1* WT or KO genotypes were stained with the anti-IRBP antibody. IRBP is localized primarily to the OS in WT animals. In *AdipoR1* KO retinas IRBP signal appears evenly distributed between the IS and OS. Scale bar = 20 µm. Z-stacks were acquired and the maximum intensity projection is displayed.

In order to assess whether a particular signaling pathway may be perturbed in the absence of ADIPOR1, we analyzed gene expression in P15 and P22 *AdipoR1* WT, HET, and KO mouse eyes by RNA-sequencing. At P22, we found more than 3300 differentially expressed genes between WT and KO samples, while at P15 only 50 were significantly different. At P22, this gene expression analysis highlighted downregulation of visual system pathways and upregulation of inflammatory and immune system markers (Supplementary Table S2). However, in the P15 gene expression dataset, we surprisingly found that *Irbp* was the most significantly upregulated gene in the *AdipoR1* KO eyes as compared to WT or HET mice. This *Irbp* upregulation decreased at P22, but still persisted on the mRNA level. We then confirmed this IRBP increase on the protein level in P15, but not P22, *AdipoR1* KO mouse eyes (Fig. 7a–c). We wondered if this IRBP increase would also be present in the *Mfrp*^*rd6*^ mouse eyes and checked a previously published microarray dataset^21^, but found no upregulation there. Yet, on the protein level we found that *Mfrp*^*rd6*^ P15 mouse eyes exhibit a significant increase in IRBP. Like the *AdipoR1* KO, by P21 IRBP protein levels in the *Mfrp*^*rd6*^ mouse eyes were similar to their cohort controls (Fig. 7d–f). Moreover, the spatial distribution of IRBP in the interphotoreceptor matrix was disrupted in the *AdipoR1* KO retinas when observed at P15 and P21 (Fig. 8). In WT retinas, IRBP was localized primarily to the OS with much lower expression in the inner segments (IS). However, in *AdipoR1* KO retinas strong IRBP signal was evenly dispersed between the IS and the OS, with no space of low signal between the OS and the ONL.

## Discussion

When ADIPOR1 and ADIPOR2 were first identified as the receptors for the metabolic hormone adiponectin^1^, these proteins generated much interest in the diabetes field with regards to their potential ability to mediate adiponectin effects on insulin response^3,22,23^. However, the role of ADIPOR1 and ADIPOR2 in controlling adiponectin effects has remained controversial due to the conflicting phenotypes observed in KO mouse models of these receptors. One published study observed a predisposition to the development of insulin resistance and diabetes for both KO models^3^, while another reported no insulin resistance in the *AdipoR1* KO and an inverse protective effect for the *AdipoR2* KO^4^ that was also observed by two other independent groups^24,25^. In the latter of these studies, *AdipoR1* KO mice were also shown to have normal insulin levels when fed a standard diet and they did not recapitulate an impaired ischemic revascularization phenotype observed previously in adiponectin-deficient mice^25^. Additionally, in a recent study of ADIPOR1 overexpression in pancreatic β-cells of WT or Akita mice, ADIPOR1 did not increase insulin secretion, reduce blood glucose, or improve β-cell survival^26^, an ascribed property of adiponectin^27–29^. Furthermore, although there have been reports of human *ADIPOR1* single nucleotide polymorphisms (SNPs) associated with diabetes and insulin resistance^30–32^, there have also been a number of opposing studies finding no such relationship^8,33–37^, while yet another group failed to validate a previously identified insulin sensitivity-associated SNP but did detect a distinct relationship between this SNP and olfaction^38,39^. In addition to these inconsistencies with regards to the metabolic contributions of *ADIPOR1* mutations, the *AdipoR1* KO has also been shown to cause retinal degeneration in mice, which was not observed in the adiponectin KO^5^. Moreover, this retinal degeneration phenotype was also observed in a zebrafish model of ADIPOR1 loss^7^, and two novel human mutations in *ADIPOR1* have been identified that cause retinitis pigmentosa^6,7^, while *ADIPOR1* mutations causing metabolic dysfunction are still lacking.

Perhaps adding to these dissonant results, is the sole reliance on mRNA levels to determine the expression pattern of these receptors^1,39,40^ as well as the many non-specific anti-ADIPOR1 antibodies used by researchers. Expression of mRNA should be considered carefully because its levels do not correlate well with protein levels^11,12^, especially when comparing different tissues^13^, and antibodies need to be rigorously validated^41^. Our antibody profile revealed that despite a number of anti-ADIPOR1 antibodies appearing in published studies, only one of these reagents could detect the endogenous protein. After identifying this specific anti-ADIPOR1 antibody, we profiled different mouse tissues for protein expression and found that unlike the previously reported ubiquitous mRNA expression pattern of *ADIPOR1* with particularly high skeletal muscle abundance^1^, the protein distribution of ADIPOR1 was much closer to a recent qRT-PCR dataset^7^, with dramatic enrichment of this protein in the eye and brain and a relative paucity of this protein in the skeletal muscle, liver, pancreas, or adipose tissue. The combination of this protein expression profile, lack of retinal degeneration in adiponectin KO mice, and the identification of human *ADIPOR1* blindness causing mutations makes a compelling argument for ADIPOR1 playing a stronger role in vision biology rather than in its more commonly proposed role in glucose metabolism.

When we explored the retinal phenotype of *AdipoR1* KO mice further, we found a number of surprising attributes. Histological and western blot analysis of protein expression in mouse and human retina tissues revealed that ADIPOR1 is expressed throughout the neural retina and the RPE with enriched expression in the OS. This contrasts with a previous report^7^ that utilized an anti-ADIPOR1 antibody that was invalidated in our study. Additional profiling of P15 and P22 *AdipoR1* WT, HET, and KO mouse eyes revealed decreased visual system proteins in the KO mice. While two week old KO animals were ostensibly normal, three week old KO animals exhibited significant retinal dystrophy as they were almost completely depleted of RHODOPSIN and displayed much lower amounts of two structural OS proteins and two phototransduction proteins.

A reduction of these key light perception components would likely explain the highly attenuated ERGs as well as the decrease of DHA, a highly OS-enriched poly-unsaturated omega-3 fatty acid^42^, previously measured in *AdipoR1* KO mice at 3–4 weeks of age^5^. Although it is possible that ADIPOR1 still contributes to DHA uptake or retention, past studies of dietary DHA deficiency have reported decreased retinal sensitivity based on ERG measurements^43–45^, but no photoreceptor loss as occurs in the *AdipoR1* KO model. Additionally, the KO of *Mfsd2a*, a major DHA transporter in the eye^46^ and brain^47^, results in drastic DHA reduction in the retina but its retinal degeneration phenotype is much less severe than that of the *AdipoR1* KO as four month old *Mfsd2a* KO mice display only minor ONL thinning and have no significant reduction in ERGs^46^.

In addition to expanding on the previously reported^5^ vital role of ADIPOR1 in the retina of young animals, we also showed that this protein is needed in adult animals. We administered AAV-*Cre* driven by either a photoreceptor or an RPE specific promoter to nine month old WT or floxed *AdipoR1* mice subretinally and observed statistically significant reduction in proteins critical for vision. Notably, although the photoreceptor-specific KO of *AdipoR1* resulted in a greater loss of ADIPOR1 compared to mice treated with the RPE promoter AAV, we detected a decrease of more retinal markers in the RPE-*Cre* treated group, suggesting a more essential role for ADIPOR1 in this cell type. We gained further support for a hypothesis of an essential ADIPOR1 RPE function by examining the *Mfrp*^*rd6*^ mouse, a mouse model of an *Mfrp* KO with striking similarities to the *AdipoR1* KO^9,19,20^. Remarkably, we found an absence of ADIPOR1 expression in the RPE layer of *Mfrp*^*rd6*^ mice compared to littermate controls, while ADIPOR1 in the adjacent photoreceptors was still present. These results suggest that analogous to our AAV-*Cre* RPE-specific *AdipoR1* KO, retinal degeneration observed with *Mfrp* mutations may actually be due to an RPE deficiency of ADIPOR1. Importantly, much like the recent ADIPOR1 reports^6,7^, *MFRP* mutations are also known to cause retinitis pigmentosa in human patients^48–50^ and it will be instrumental to assess whether their disease is caused by ADIPOR1 loss and whether it can be prevented via rescue of ADIPOR1 expression.

When we further analyzed *Mfrp*^*rd6*^ eyes by western blotting, we observed that while whole WT eyes displayed a distinct doublet of ADIPOR1 signal, *Mfrp*^*rd6*^ eyes appeared to only have a single band. An additional comparison of this whole mouse eye ADIPOR1 running pattern to a pure RPE sample showed that the signal doublet can be broken down into a top RPE band and bottom non-RPE band, likely representing the neural retina. Importantly, congruent to our IHC results, *Mfrp*^*rd6*^ eyes were missing only the top band, the inferred RPE signal. We also noticed slightly different ADIPOR1 running patterns in the other mouse tissues: a single band in the brain, heart, and skeletal muscle, and distinct doublets in the eye and liver. We speculate that these differences are either splice isoforms of *AdipoR1*^51^ or post-translational modifications that may modify ADIPOR1 function. However, the mouse genome does not contain alternative transcripts encoding a protein of a different molecular weight and of the four predicted human protein coding transcripts only the canonical encoded protein sequence is predicted to run near the molecular weight we observed (Ensembl genome browser 91, December 2017)^52^. It will be intriguing to ascertain whether these seemingly different forms of ADIPOR1 do indeed modify its function leading to distinct molecular properties.

In an effort to better understand the mechanism of *AdipoR1* KO induced retinal degeneration, we performed RNA-sequencing analysis in two and three week old animals. While the three week old animals highlighted many known gene expression changes that have been observed in a number of other retinal degeneration models^53–55^, namely a downregulation in visual perception pathways and an upregulation in inflammation and immune system response genes, as would be expected if the photoreceptors were starting to die at this age, we were surprised to detect a unique conspicuous feature of the *AdipoR1* KO at two weeks of age – an upregulation of *Irbp*, a gene that encodes a key retinoid transport protein produced and secreted exclusively by photoreceptors. We confirmed this IRBP upregulation on the protein level in two week old animals, and found that similar to the observed mRNA changes, IRBP was reduced back to normal levels at three weeks of age. Intriguingly, protein analysis of *Mfrp*^*rd6*^ mice showed the same IRBP expression pattern with an early peak followed by normalization. Notably, *Irbp* KO mice exhibit progressive retinal degeneration^56^ and multiple animal models of retinal degeneration have also observed a decrease in IRBP expression^57–59^, therefore the sharp increase in this protein in a retinal degeneration model is surprising. However, one other retinal degeneration model has been described where an increase in IRBP was also detected, the vitiligo mouse model^60–62^. Similarly to the *AdipoR1* KO and *Mfrp*^*rd6*^, vitiligo mice develop a progressive retinal degeneration accompanied by an increase in IRBP protein levels^60^ as well as an IRBP mislocalization^63^ from the OS to the IS that we also observed. Due to this phenotypic mimicry and since the vitiligo mouse model appears to have a dysfunction in retinoid metabolism^60,64^, this data presents an exciting hypothesis for whether ADIPOR1 functions to control retinoid levels as well. Additionally, recent data has demonstrated that ADIPOR1 is likely a ceramidase^28,65,66^ and ceramide signaling has been proposed to function in maintaining photoreceptor survival in flies, mice, and humans^67^. Moreover, another enzyme, dihydroceramide desaturase-1 (DES1), has also recently been reported to function as a retinol isomerase in the cone visual cycle^68^, suggesting that at least one other enzyme can have activity on both, ceramides and retinoids. Taken together, the enrichment of ADIPOR1 in the retina combined with its retinitis pigmentosa causing mutations, described ceramidase activity, and dysregulation of IRBP, appears to point toward a pivotal role in the visual cycle that requires further exploration.

## Materials and Methods

All experiments used whole eyes unless otherwise indicated.

### Animals

All procedures and housing conditions were approved and performed as described in the associated Novartis Cambridge Institutional Animal Care and Use Committee protocol. *AdipoR1* KO (B6;129S5 Adipor1 <tm1.2Lex>) and floxed (B6;129S5 Adipor1 < tm1.1Lex>) mice were purchased from Taconic Biosciences (Germantown, NY). Mouse breeding was set up as a HET by HET mating to reduce the chance of any maternal effects arising from the *AdipoR1* KO. Genotyping was assessed by PCR according to Taconic recommendations. Primers 5′-CAGGCTGGCCTCGAGTTCAG-3′ and 5′-ATGGACAAATTCCTTGGCAAG-3′ were used to amplify a 370 bp KO band and primers 5′-CAGGCTGGCCTCGAGTTCAG-3′ and 5′-GCCAGCTCCACTGTGTCAGC-3′ were used to amplify a 389 bp WT band. A KO genotype was confirmed by presence of the KO band alone, WT genotype produced WT band alone, HETs produced a KO and a WT band. All breeder mice were tested and confirmed to be negative for the *rd1* and *rd8* mutations. Mixed gender mice were used for all experiments.

*Mfrp*^*rd6*^ (B6.C3Ga- *Mfrp*^*rd6*^/J, Stock No. 003684) and control B6 (C57BL/6 J, Stock No. 000664) (WT) mice in this study were bred and maintained in standardized conditions of the Production and Research Animal Facilities at The Jackson Laboratory (JAX). They were provided with a NIH31 6% fat chow diet and acidified water, in a pathogen-free vivarium environment with a 14-hour light/10-hour dark cycle. All experiments were approved by the Institutional Animal Care and Use Committee and conducted in accordance with the ARVO Statement for the Use of Animals in Ophthalmic and Vision Research.

### OCT imaging

Retinal images were acquired using a spectral domain optical coherence tomography system (Envisu R-Class, Bioptigen, Morrisville, NC). Images were acquired approximately one week after injection to exclude any eyes with substantial complications from the injection procedure. OCT imaging for retinal thickness assessment occurred at 4 and 13 weeks post-injection. Prior to the OCT procedure, animals were anesthetized with IP ketamine/xylazine cocktail and received 1.0% cyclopentolate and 2.5–10% phenylephrine topically for dilation in addition to 0.5% proparacaine as a topical anesthetic. Animals were placed on a heating pad during image acquisition and eye hydration was maintained with topical application of mild to moderate GenTeal drops (Novartis). A high quality image of the retina was generated by aligning and averaging 20 horizontal (nasal-temporal) b-scans centered on the optic nerve. Scans were 1.7 mm in length and consisted of 1000 a-scans/b-scan. Image co-registration and averaging was performed using MATLAB (Mathworks, Natick, MA). A custom algorithm in MATLAB was subsequently used to manually delineate the inner limiting membrane and the basal RPE at Bruch’s membrane. Retinal thickness was reported as the average distance between the two lines across the visible portion of the retina with the exception of the central 340 μm of the b-scan that was excluded due to variable retinal thickness near the optic nerve.

### AAV virus generation and subretinal injection

AAV expression plasmids encoding *GFP-Cre* driven by either *CMV*, human *VMD2*^69^, or human *IRBP*^70^ were generated to express *Cre* constitutively, in the RPE^69^, or in photoreceptors^70^, respectively. AAV vectors were produced at the University of Massachusetts Medical School Gene Therapy Center and Vector Core using these plasmids. The *CMV-GFP-Cre* construct was packaged into AAV serotype 1 while the *VMD2* and *IRBP* constructs were packaged into AAV serotype 2. AAV Cre activity was confirmed in HEK293-loxP-GFP-RFP (Cat#: SC018-Puro, GenTarget Inc). Both eyes of 9 month old animals were injected subretinally as previously described^71^ at a dose of 1 × 10^9 genome copies per eye. Mice were taken down 5 months post injection for dissection and western blot analysis. Floxed B6;129S5 Adipor1 <tm1.1Lex> mice of either WT or unexcised KO were injected. Uninjected animals served as naïve age matched controls.

### Human tissues preparation

All human samples were collected with the approval of the Novartis HTN (human tissue network), following the guidance of OPH HTN with informed consent from all donors. Human eye tissues were fixed at room temperature with Modified Davidson’s Fixative for two days and followed by 70% ethanol for another two days. After fixation, the tissues were processed and embedded in paraffin. The paraffin embedded eye sections were cut at 5 µm and used for *ADIPOR1* mRNA detection and the expression of ADIPOR1 and MFRP proteins. For western blotting, unfixed human eye tissue was dissected and frozen prior to lysis.

### *In situ* hybridization – RNAscope

Visualization of *ADIPOR1* transcript variant 1 mRNA (RNAscope® 2.5 LS Probe - Hs-ADIPOR1 (Cat# 472298, ACD) was done in Automated Leica Bond RX according to manufacturer’s instruction for RNAscope® 2.5 LS (Advanced Cell Diagnostics, Newark, CA, USA). RNAscope ® has been shown to be capable of single mRNA molecule detection. The positive control probe consisted of a proprietary probe for POLR2A (Cat# 310458), while the negative control probe targeted dapB of Bacillus subtilis (Cat # 312038).

### Immunohistochemistry

#### Human samples

Detection of ADIPOR1 and MFRP proteins was done in Automated Leica Bond RX. Anti-ADIPOR1 antibody (Cat# 18993, IBL) was applied at 1:50 dilution and detected by Bond Polymer Refine Red Detection (Leica Biosystems, Cat# DS9390); anti-MFRP antibody (Cat # AF1915, R&D Systems) was applied at 1:1000 dilution and detected by Bond Polymer Refine Detection (Cat # DS9713, Leica Biosystems) with Vina Green^TM^ Chromogen (Cat # BRR807A, Biocare Medical).

#### Mouse samples

*AdipoR1* mouse model whole eyes of age P15 or P21 were harvested and immersed in ice cold 3.2% PFA made in PBS (pH = 7.4) for 30 minutes. The eyes were washed with PBS and left in cryoprotective solution (20% sucrose in PBS) overnight at 4 °C and then embedded in OCT (Optimal Cutting Temperature compound, Cat # 62550-01, Electron Microscopy Sciences) and frozen. Frozen cryosections were cut at 10-16 μm thickness. Sections were blocked with 10% Normal Goat Serum (NGS, Cat # 5425, Cell Signaling Technology, Danvers, MA) with 0.01% Triton-X-100 in PBS for 1 hour at RT. Primary antibodies were diluted in 10% NGS, 0.01% Triton-X-100, PBS and incubated with the sections overnight at RT in a humidified chamber. Sections were washed with PBS and incubated with the secondary antibody (Goat anti-Rabbit Alexa-647 at 1:1000 dilution, Cat # A-21245, Thermo Fisher Scientific) for 45 minutes at RT in 10% NGS, 0.01% Triton-X-100, PBS. After secondary antibody washing, sections were stained with Hoechst 33342 (1:10,000 dilution, Cat # H3570, Thermo Fisher Scientific) for 10 min at RT, washed, and sealed with Aqua-Poly/Mount (Cat # 18606-20, Polysciences, Inc.). Fluorescence images were acquired using the LSM 880 confocal microscope with Airy Scan (Zeiss). Exposure settings for image acquisition were set to KO control levels or no primary controls.

For mouse IHC utilizing paraffin, mouse eyes were collected and fixed in 10% neutral buffered formalin for 2 days and paraffin embedded, sectioned at 5 μm thickness and mounted onto slides (Superfrost® Plus, Fisher Scientific, Waltham, MA). IHC was performed using the Leica Bond Rx IHC/ISH slide staining system. Slides were treated by Epitope Retrieval #2 (AR9640) for 20 minutes and incubated with primary antibodies (anti-ADIPOR1, Cat# 18993, IBL at 1:200 or anti-MFRP, Cat# AF3445, R&D Systems at 1:250) for 30 minutes; then were incubated with Bond Polymer AP for 20 minutes and Bond Polymer Refine Detection (Ds9800) for 12 minutes. Control sections were only incubated with anti-IgG and stained. Finally, stained slides were covered and scanned into Aperio AT2 slide scanner (Leica).

### RNA-sequencing analysis

*AdipoR1* WT, HET, and KO whole mouse eyes of age P15 or P22 were collected and stored in RNAlater™ Stabilization Solution (Cat # AM7020, Thermo Fisher Scientific) at −80 °C. Four biological replicates were used per genotype of each age (P15 and P22) with both eyes collected for one independent sample. For RNA extraction, the eyes were thawed and lysed per manufacturer instructions using the RNeasy Mini Kit (Cat # 74106, Qiagen) with on column DNase I digestion (Cat # 79254, Qiagen). RNA concentration was measured using Nanodrop 2000 (Thermo Fisher Scientific) and RNA integrity was confirmed using the Bioanalyzer 2100 (Agilent). RNA-Seq libraries were constructed using a stranded Poly(A) + selection protocol and sequenced by paired-end chemistry on the Illumina HiSeq. 2500 platform. Technical quality control (QC) was performed on the RNA-Seq libraries utilizing RNASeQC^72^. RNA-Seq paired-end short reads were aligned to the GENCODE mouse reference genome (GRCm38.p4) using STAR (v2.4.1a)^73^, with default settings. Transcript quantification was conducted with RSEM (v1.2.22)^74^, resulting in gene counts and normalized transcripts per million (TPM). Custom R scripts were used for biological QC and differential gene expression. Specifically, the edgeR and limma packages were applied to determine significantly differentially expressed genes between experimental groups^75^. A gene was defined as differentially expressed if it had an adjusted p-value ≤ 0.05.

### Cell culture: HEK293T cells

HEK293T cells (Cat # CRL-3216™, ATCC®, Manassas, VA) were maintained in DMEM/F-12, GlutaMAX™ (Cat # 10565042) + 10% Fetal Bovine Serum (FBS), certified, heat inactivated (Cat # 10082147), 1% MEM Non-Essential Amino Acids (Cat # 11140050), 1% Sodium Pyruvate (Cat # 11360070), 1% Antibiotic-Antimycotic (Cat # 15240062) – all media components were from Thermo Fisher Scientific.

To generate exogenous ADIPOR1 expressing cells, HEK293T cells were plated as 3 million cells per 10 cm plate the day before transfection, and transfected using 0.5 µg of Flag-ADIPOR1 expression plasmid (Cat # HG14109-NF, Sino Biological, Beijing, China) using Lipofectamine 3000 (Thermo Fisher Scientific) and Opti-MEM™ (Thermo Fisher Scientific). Cells were harvested for lysis 48 hours after transfection.

To generate *ADIPOR1* KO and CRISPR control cells, HEK293T were transfected with plasmids encoding Cas9-2A-Puro + gRNA carrying gRNAs targeting the first coding exon of *ADIPOR1* or control gRNAs that were not predicted to have matches in the human genome.

*ADIPOR1* gRNAs 5′−3′:

GGAAGCTGACACGGTGGAAC

AGCCAGATGTCTTCCCACAA

Non-targeting gRNAs 5′−3′:

GCACTCACATCGCTACATCA

GCACTACCAGAGCTAACTCA

HEK293T cells were plated as 500,000 cells per well of a 6 well plate and transfected with 0.6 µg of total DNA encoding either two plasmids targeting *ADIPOR1* or two non-targeting plasmids (0.3 µg per vector) with 1.2 µL of P3000 and 5 uL of Lipofectamine 3000 in a mix of 200 µL of total Opti MEM. At 48 hours post transfection, the cells were selected with 5 µg/mL puromycin for 4 days, a treatment that killed all WT untransfected cells. The KO cells were then split as single cells using TrypLE™ Express Enzyme (Cat # 12605036, Thermo Fisher Scientific) and plated at a low density to form colonies from individual cells. Five of the six picked clones tested negative for ADIPOR1 by western blotting (Cat # 18993, IBL). Further KO validation was performed by DNA sequencing (Genewiz, South Plainfield, NJ) of the *ADIPOR1* gene on clones 4 and 5 (see Supplementary Fig. 1d, lanes 4 and 5) and KO was confirmed on the DNA level. These two KO clones were used to profile all anti-ADIPOR1 antibodies. All HEK293T cells were plated as 3 million per 10 cm plate and expanded for 48 hours before cell lysis.

### Cell culture: H9-RPE cells

H9 human embryonic stem cells (hESCs -WiCell, Madison, WI) were differentiated to RPE cells using the previously published protocol^76^ and frozen for future use. To generate H9-RPE cell lysate, cells were thawed and plated as 5 million cells per well of a 6 well plate in RPE media (RtEGM™ BulletKit®, Cat # 00195406 and 00195407, Lonza, Basel, Switzerland). Cells were fed with fresh media daily until they formed a confluent monolayer and then they were fed with RPE media without FBS every other day for 3 months until cell lysis for protein analysis.

### Statistical analysis

One-way analysis of variance (ANOVA) (α = 0.05) with Dunnett’s multiple comparisons test (WT set as control) or Tukey’s multiple comparisons test, or an unpaired two tailed t-test were used as indicated. Statistics were analyzed in GraphPad Prism7.

### Western blot

Cells were lysed with Cell Lysis Buffer (Cat # 9803, Cell Signaling Technology, Danvers, MA) containing Protease/Phosphatase Inhibitor Cocktail (Cat # 5872, Cell Signaling Technology, Danvers, MA) on ice for 30 min with intermittent pipetting. Tissues were lysed in RIPA Buffer (Cat # 9806, Cell Signaling Technology, Danvers, MA) containing the same Protease/Phosphatase Inhibitor Cocktail. Following dissection, tissues were placed into lysis buffer on ice, combined with a stainless steel bead (Cat # 69989, Qiagen), and homogenized using a TissueLyser II (Cat # 85300, Qiagen) in a cold room for 2 min at a frequency of 30 Hz. Following homogenization, tissue samples were briefly sonicated (XL-2000, Misonix). Upon lysis completion, cell and tissue lysate was centrifuged at 20,000xg for 25 min at 4 °C. The soluble fraction was kept for further analysis and quantified using the BCA assay (Cat # 23225, Thermo Fisher Scientific). Quantified protein samples were combined with Laemmli Sample Buffer (Cat # 1610747 A, Bio-Rad) containing 10% β-mercaptoethanol (BME), heated to 37 °C for 10 minutes or otherwise indicated temperature, and loaded onto precast gels - AnykD™ (Cat # 5671125, Bio-Rad) or 4–20% (Cat # 5671095, Bio-Rad). We found that the AnykD gels were best for resolving the ADIPOR1 eye signal doublet. Gels were ran on a Criterion™ Cell system (Bio-Rad). Gel transfer onto nitrocellulose membranes (Cat # IB23001, Thermo Fisher Scientific) was performed using the iBlot 2 system (Cat # IB21001, Thermo Fisher Scientific). Blots were blocked with 5% non-fat dry milk (NFDM, Cat # 9999, Cell Signaling Technology, Danvers, MA) in PBS for 1 hour at room temperature (RT). Blots were incubated with primary antibodies in 0.05% Tween-20 (Cat # 161–0781, Bio-Rad) with 5% NFDM in PBS overnight at 4 °C. After primary antibody incubation, blots were washed with PBS + 0.05% Tween-20 and incubated with secondary HRP-coupled antibodies for 1 hour at RT. After secondary antibody washing, blots were developed using Clarity™ Western ECL Substrate (Cat # 1705061, Bio-Rad) and imaged using the FluorChem M system (ProteinSimple). Precision plus dual color protein ladder (Cat # 1610374, Bio-Rad) was used to assess molecular weight. For list of antibodies used see Supplementary Table S1.

For ADIPOR1 protein analysis across different mouse tissues, adult mouse tissue protein lysate from C57BL/6 J mice was used (Cat #: MT-101-C57 skin, MT-102-C57 skeletal muscle, MT-103-C57 adipose tissue, MT-106-C57 eye, MT-313-C57 pancreas, MT-314-C57 liver, MT-201-C57 brain, MT-801-C57 heart, MT-901-C57 kidney - Zyagen, San Diego, California). Zyagen brain samples contained whole brain tissue lysate while brain tissue from WT and KO *AdipoR1* animals was dissected as a piece of the cerebral cortex. No specific region of the cerebral cortex was collected. A total of 40 µg of protein was loaded per lane. Post western blot analysis, Amido Black staining solution (Cat # A8181, Sigma) was used to verify equal transfer and to assess protein levels.

## Electronic supplementary material

Supplementary Information

Table S1

Table S2

## Data Availability

All data generated or analyzed during this study are included in this article, and its Supplementary Information files.
