# Supplementary material for: ADIPOR1 is essential for vision and its RPE expression is lost in the Mfrprd6 mouse
Source: Sci Rep. 2018 Sep 25;8:14339. doi: 10.1038/s41598-018-32579-9 (PMC6156493; doi:10.1038/s41598-018-32579-9)

Supplementary Information File

Title: ADIPOR1 is essential for vision and its RPE expression is lost in the *Mfrp*^{rd6} mouse

Authors: Valentin M. Sluch[1*], Angela Banks[1], Hui Li[1], Maura A. Crowley[1], Vanessa Davis[2], Chuanxi Xiang[1], Junzheng Yang[1], John T. Demirs[1], Joanna Vrouvlianis[1], Barrett Leehy[1], Shawn Hanks[1], Alexandra M. Hyman[1], Jorge Aranda[1], Bo Chang[3], Chad E. Bigelow[1], Dennis S. Rice[1*]

1. Department of Ophthalmology, Novartis Institutes for BioMedical Research, Cambridge, Massachusetts, United States
2. Global Scientific Operations, Novartis Institutes for BioMedical Research, Cambridge, Massachusetts, United States
3. The Jackson Laboratory, Bar Harbor, Maine, United States

***Authors for correspondence:**

Valentin M. Sluch, Department of Ophthalmology, Novartis Institutes for BioMedical Research, 22 Windsor Street, Cambridge, MA 02139, USA; valsluch@gmail.com.

Dennis S. Rice, Department of Ophthalmology, Novartis Institutes for BioMedical Research, 22 Windsor Street, Cambridge, MA 02139, USA; dennis.rice@novartis.com.

Supplementary Figure-1. Heat sensitivity of ADIPOR1 protein and generation of HEK293T *ADIPOR1* KO clones for antibody validation

(**a**) Exogenous ADIPOR1 protein is heat sensitive. Flag-ADIPOR1 transfected HEK293T lysate was used. Lanes = samples were heated for 10 min at the following temperature: 1 – RT, 2 – 37 °C, 3 – 60 °C, 4 – 95 °C. (**b**) Endogenous ADIPOR1 protein is heat sensitive. Untransfected HEK293T lysate was used. Lanes = samples were heated for 10 min at the following temperature: 1 – RT, 2 - 37°C, 3 - 60°C, 4 - 95°C. (**c**) Effect of short heat exposure on endogenous ADIPOR1 protein. Untransfected HEK293T lysate was used. Lanes = samples were heated for: 1 - 10 min at 37°C, 2 - 1 min at 95°C, 3 - 2 min at 95°C, 4 - 3 min at 95°C, 5 - 5 min at 95°C, 6 - 10 min at 95°C (**d**) Generation of HEK293T *ADIPOR1* KO clones. Lanes: 1 = WT HEK293T, 2-7 = individual picked clones post *ADIPOR1* CRISPR KO treatment. Clones 4 and 5 (lanes 4 and 5) were used for all antibody validation. Western blots: (a) anti-Flag, (b-d) IBL-18993 anti-ADIPOR1; CYCLOPHILIN B used as loading control. Each membrane was cut and probed separately for the analyzed protein and the loading control.

Supplementary Figure-2. Antibody profiling in HEK293T

Full length blots of 15 different anti-ADIPOR1 antibodies are shown. CYCLOPHILIN B was used as a loading control. Lanes: 1 – Flag-ADIPOR1 transfected HEK293T, 2 – WT/Untransfected HEK293T, 3 – CRISPR negative control transfected HEK293T, 4 – *ADIPOR1* KO HEK293T Clone 4, *ADIPOR1* KO HEK293T Clone 5. The IBL-18993 antibody capable of detecting both endogenous and exogenous ADIPOR1 is labeled in red text, while antibodies that detected exogenous ADIPOR1 but failed to detect endogenous protein are labeled

in blue text. ADIPOR1 runs near 37 kDa. OE=overexpression. Each membrane was cut and probed separately for the analyzed protein and the loading control.

Supplementary Figure-3. Full length images of western blots from Figure-1b,c

(**a**) ADIPOR1 protein expression profile from different tissues. Lanes: 1 - Adipose tissue, 2 – Brain, 3 – Eye, 4 – Heart, 5 – Kidney, 6 – Liver, 7 – Pancreas, 8 - Skeletal muscle, 9 – Skin, 10 - P22 *AdipoR1* WT mouse eye, 11 - P22 *AdipoR1* KO mouse eye. Lanes 1-9 are from adult mouse tissue. Equal amounts of protein were loaded. An anti-ADIPOR1 western blot and an amido black total protein stain are displayed. (**b**) ADIPOR1 western blots from different tissues ran separately. VINCULIN was used as a loading control. Four and a half month old *AdipoR1* WT and KO mice were used for tissue collection. Each lane represents a sample from an individual mouse. Each membrane was cut and probed separately for the analyzed protein and the loading control.

Supplementary Figure-4. ADIPOR1 is expressed in the human neural retina and RPE

(**a**) RNAScope ISH on human tissues for *ADIPOR1* mRNA or a negative control. (**b**) IHC for ADIPOR1 protein or a negative control in paraffin embedded human retina sections. ADIPOR1 is observed via the red reaction product (**c**) Western blots for ADIPOR1, RHODOPSIN (photoreceptor marker), and RPE65 (RPE marker) using dissected human eye tissue of neural retina or posterior eye cup containing the RPE. β-ACTIN was used as a loading control. ADIPOR1 protein is present in both, retina and RPE containing fractions. The membrane was cut and probed separately for the analyzed proteins and the loading control. ADIPOR1 was probed on a separate membrane. (**d**) IHC for MFRP protein or a negative control in paraffin embedded

human retina sections. MFRP is observed via the green reaction product. Scale bar = 20 μm for (a,b,d).

Supplementary Figure-5. OCT retinal thickness measurement

OCT was used to measure total retinal thickness at 4 and 13 weeks post AAV-Cre injection. No matched pair of WT to Floxed samples showed statistically significant differences using ANOVA ($\alpha = 0.05$) with Tukey's multiple comparisons test with each eye treated as an independent biological replicate.

Supplementary Figure-6. Comparison of naive eyes from 13 months old mice

(**a**) Western blot for RHODOPSIN and CRX of naive, uninjected mouse eyes is shown. VINCULIN was used as a loading control. Each lane represents an individual eye from a mouse of that genotype, n=10 for WT, n=9 for Floxed. Each membrane was cut and probed separately for the analyzed protein and the loading control. (**b**) Densitometry quantification for RHODOPSIN and CRX from (a). No significant differences between naïve WT and floxed mice were found. Unpaired two tailed t-test was used. *=p<0.05, **=p<0.01, ***=p<0.001, ****=p<0.0001, NS=not significant. Error bars represent standard deviation.

P values: RHODOPSIN p=0.9794, CRX p=0.2642

Supplementary Figure-7. AAV *CMV-Cre* treated adult mice western blot analysis of retinal markers

(**a**) Western blots of *CMV-Cre* treated mouse eyes are shown. VINCULIN or α-TUBULIN was used as a loading control. Each lane represents an individual eye from a mouse of that genotype,

n=10 for WT, n=8 for Floxed. Each membrane was cut and probed separately for the analyzed proteins and the loading control. (**b**) Densitometry quantification of blots in (a). Unpaired two tailed t-test was used. *=p<0.05, **=p<0.01, ***=p<0.001, ****=p<0.0001, NS=not significant. Error bars represent standard deviation.

CMV group - P values: ADIPOR1 - p=0.0008; RHODOPSIN - p=0.0624; CRX - p=0.1969; RPE65 - p=0.0016; GNAT1 - p=0.3627; IRBP - p=0.2313

Supplementary Figure-8. AAV *IRBP-Cre* treated adult mice western blots

Western blots for visual system proteins in *IRBP-Cre* treated mouse eyes are shown. VINCULIN or α-TUBULIN was used as a loading control. Each membrane was cut and probed separately for the analyzed proteins and the loading control. Densitometry quantification of this figure is shown in Figure-5a.

Supplementary Figure-9. AAV *VMD2-Cre* treated adult mice western blots

Western blots for visual system proteins in *VMD2-Cre* treated mouse eyes are shown. VINCULIN or α-TUBULIN was used as a loading control. Each membrane was cut and probed separately for the analyzed proteins and the loading control. Densitometry quantification of this figure is shown in Figure-5b.

Supplementary Figure-10. *AdipoR1* KO mice express MFRP

IHC on P21 *AdipoR1* WT, HET, or KO mouse eyes is shown. Anti-MFRP staining was performed on the indicated *AdipoR1* genotype eye. MFRP is observed via the red reaction product. *AdipoR1* KO mice do not lack MFRP. Scale bar = 20 µm.

Supplementary Table S1. List of antibodies used in this study

Supplementary Table S2. Gene expression profile of *AdipoR1* WT, HET, and KO mice

Gene expression of *AdipoR1* WT, HET, and KO mice of P15 and P22 days of age was analyzed and compared between the different genotypes. Differentially expressed genes in order of statistical significance are displayed.

Supplementary Figure-1

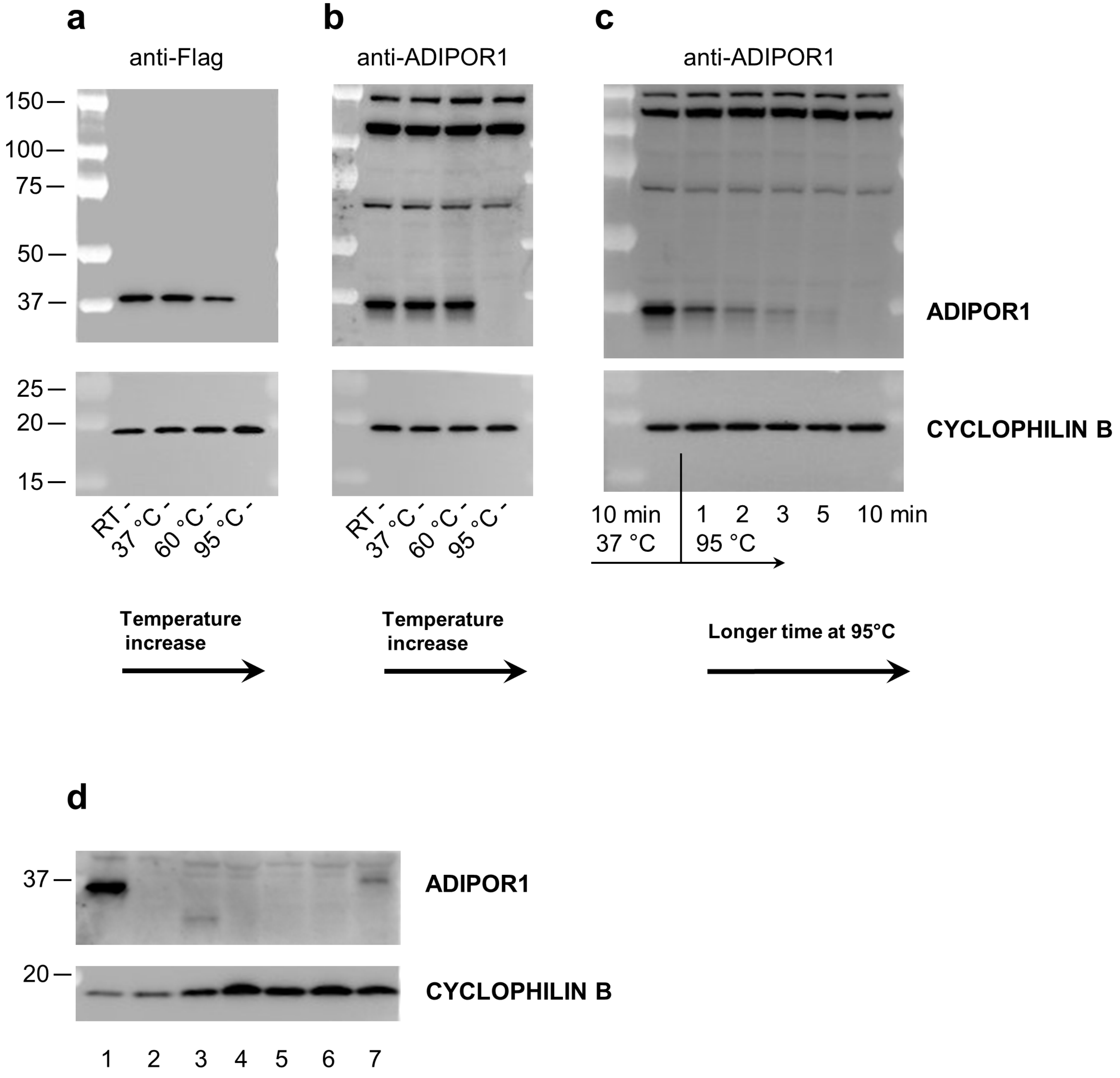

Supplementary Figure-2

anti-ADIPOR1 western blots; CYCLOPHILIN B used as loading control

Lanes: 1, 2, 3, 4, 5 → OE, WT, CRISPR-CTRL, KO-1, KO-2

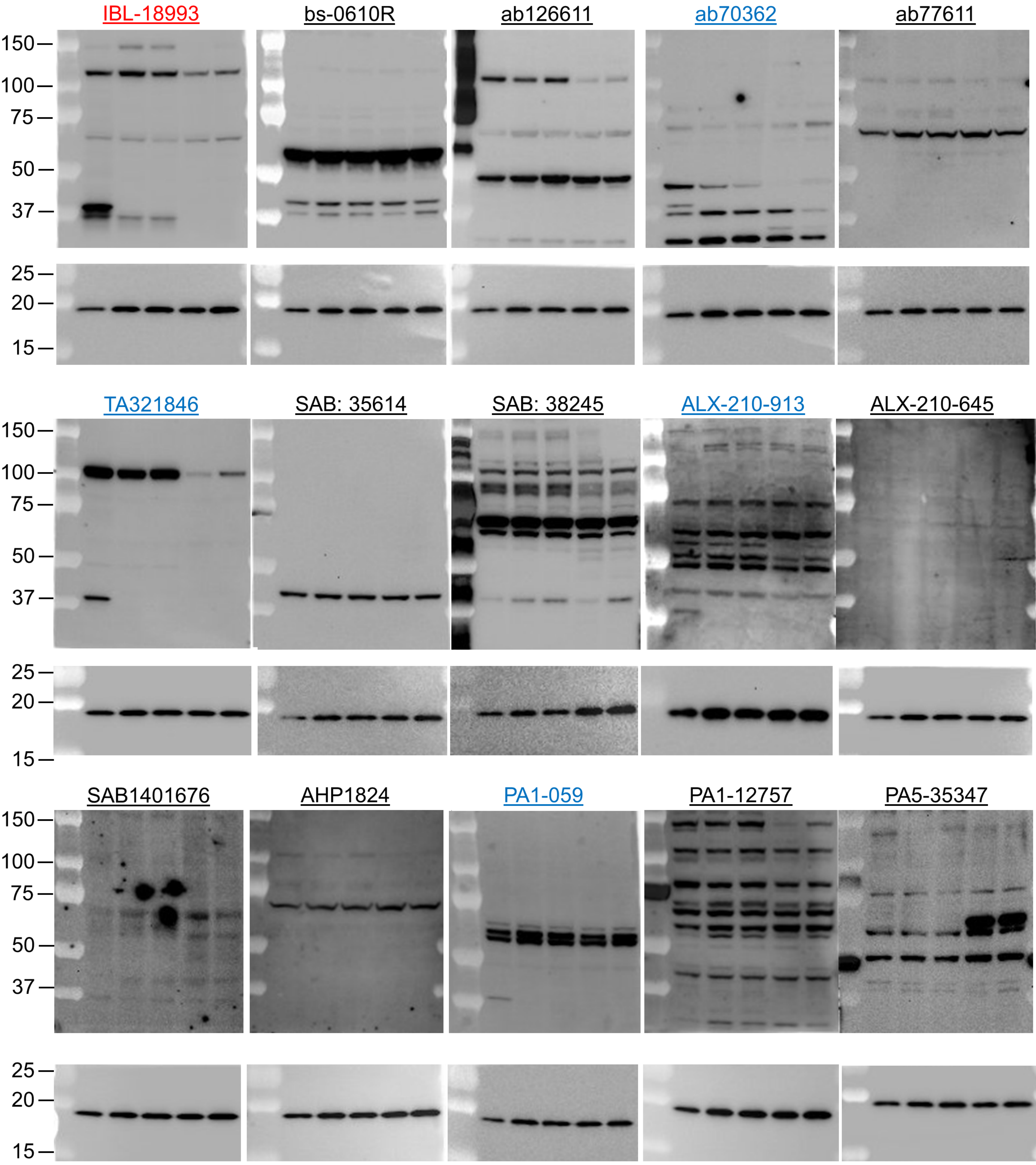

Supplementary Figure-3

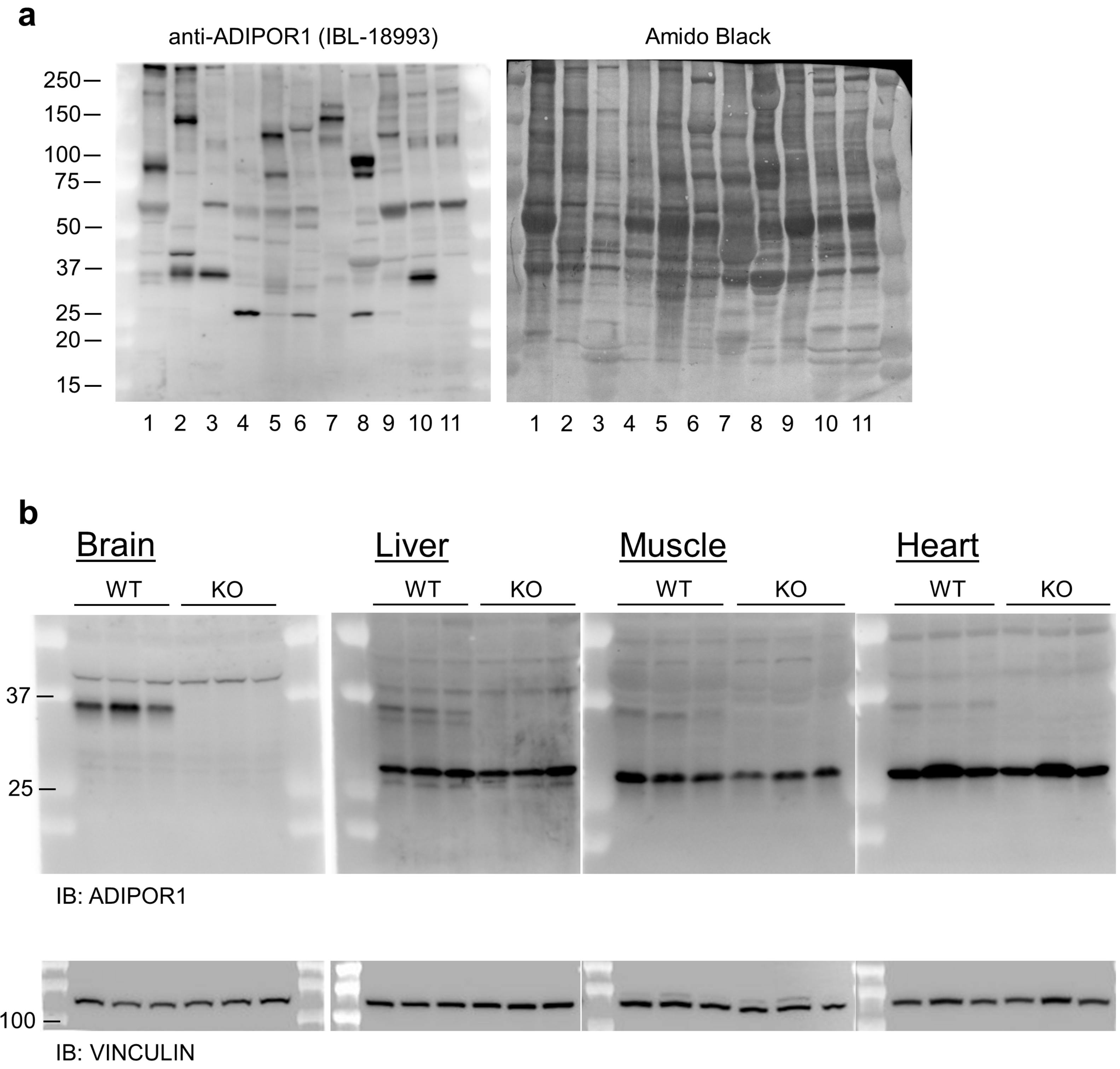

Supplementary Figure-4

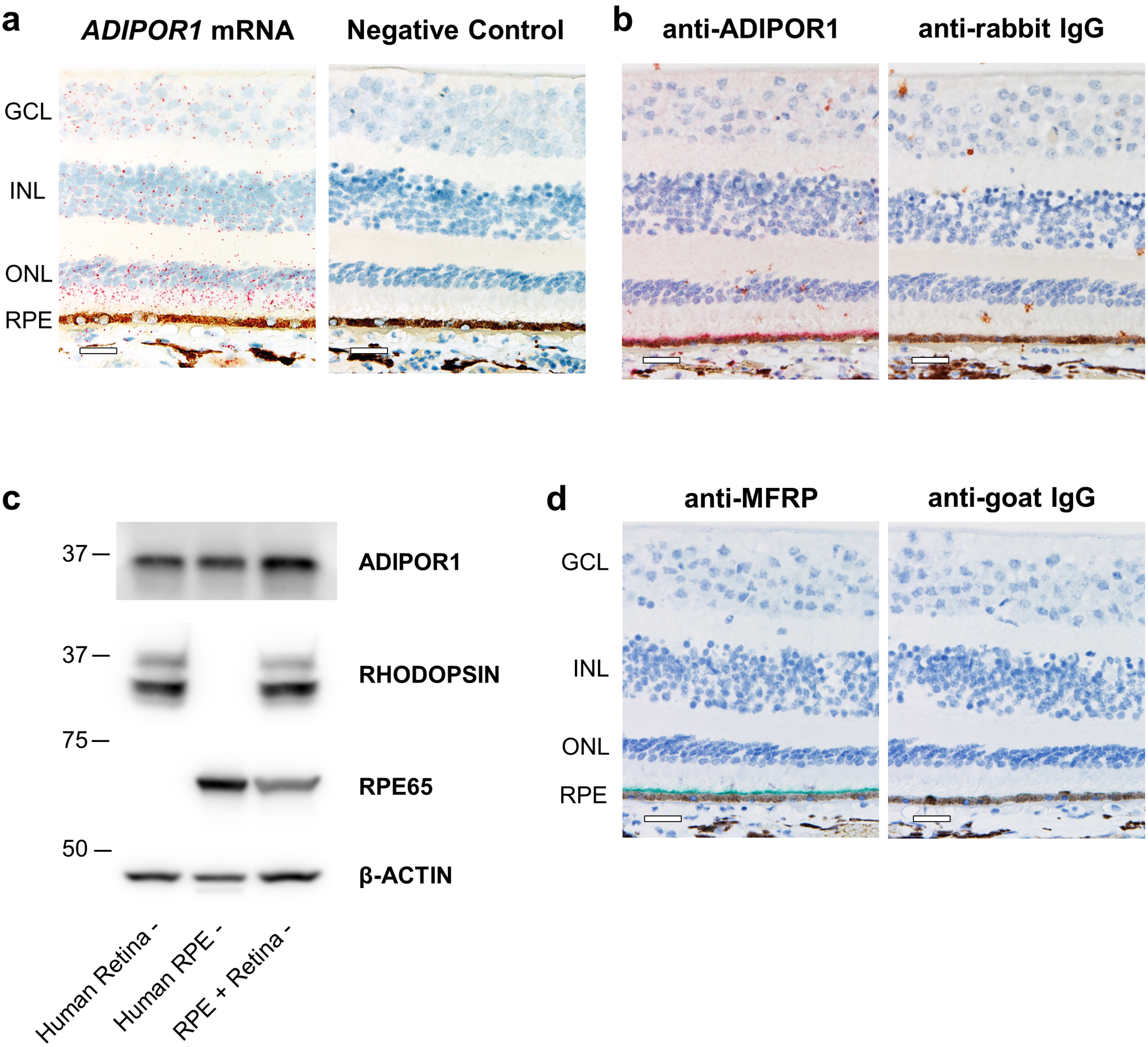

Supplementary Figure-5

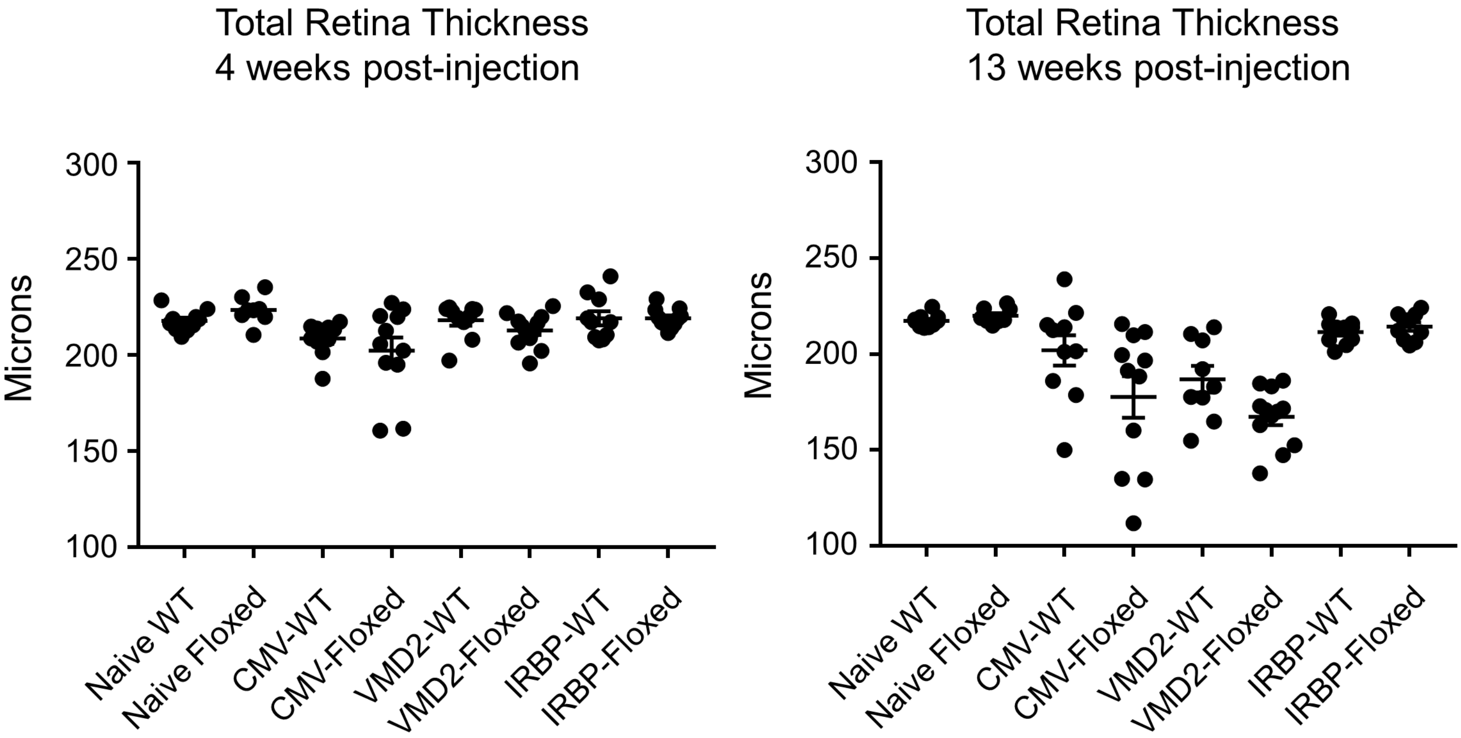

Supplementary Figure-6

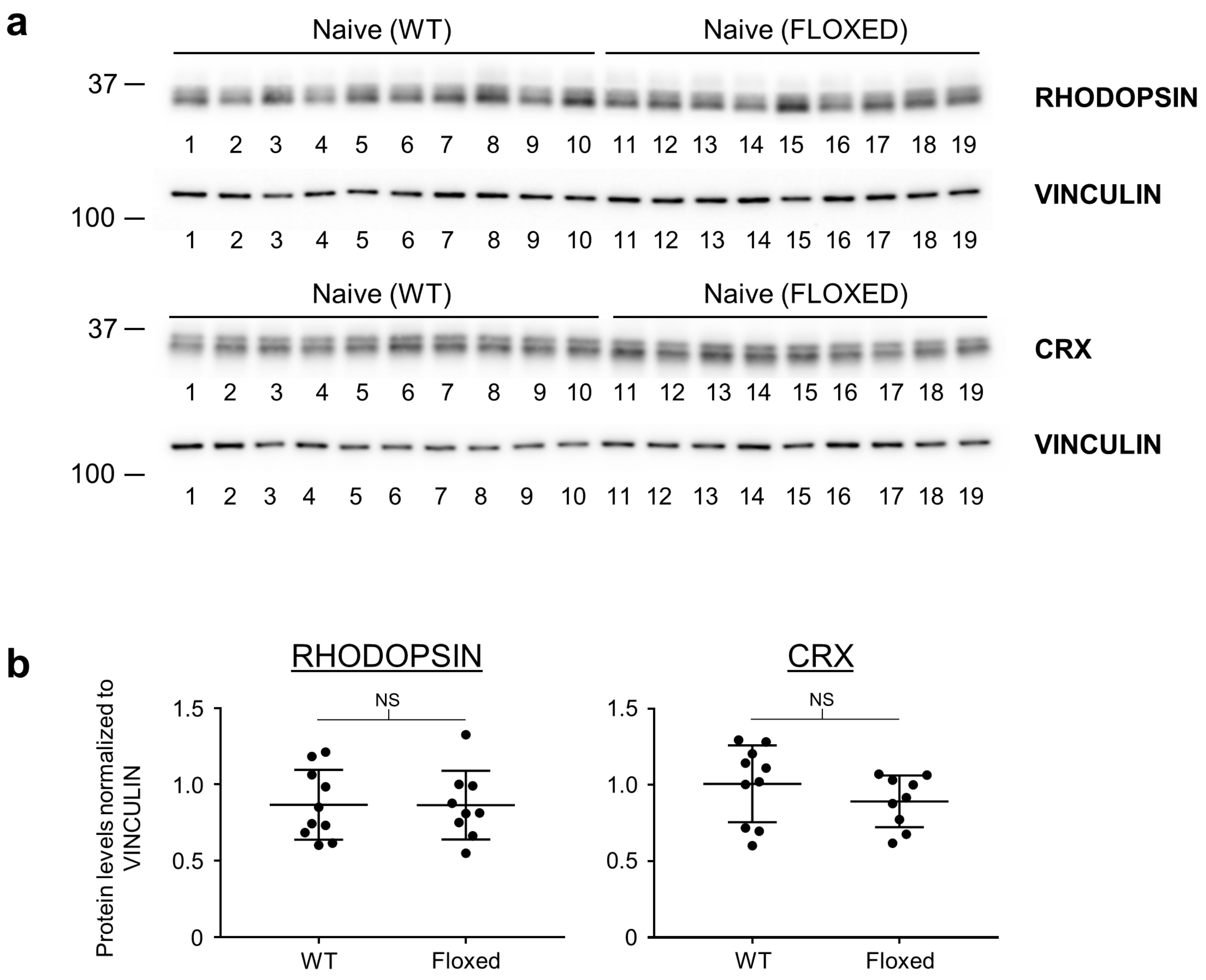

Supplementary Figure-7

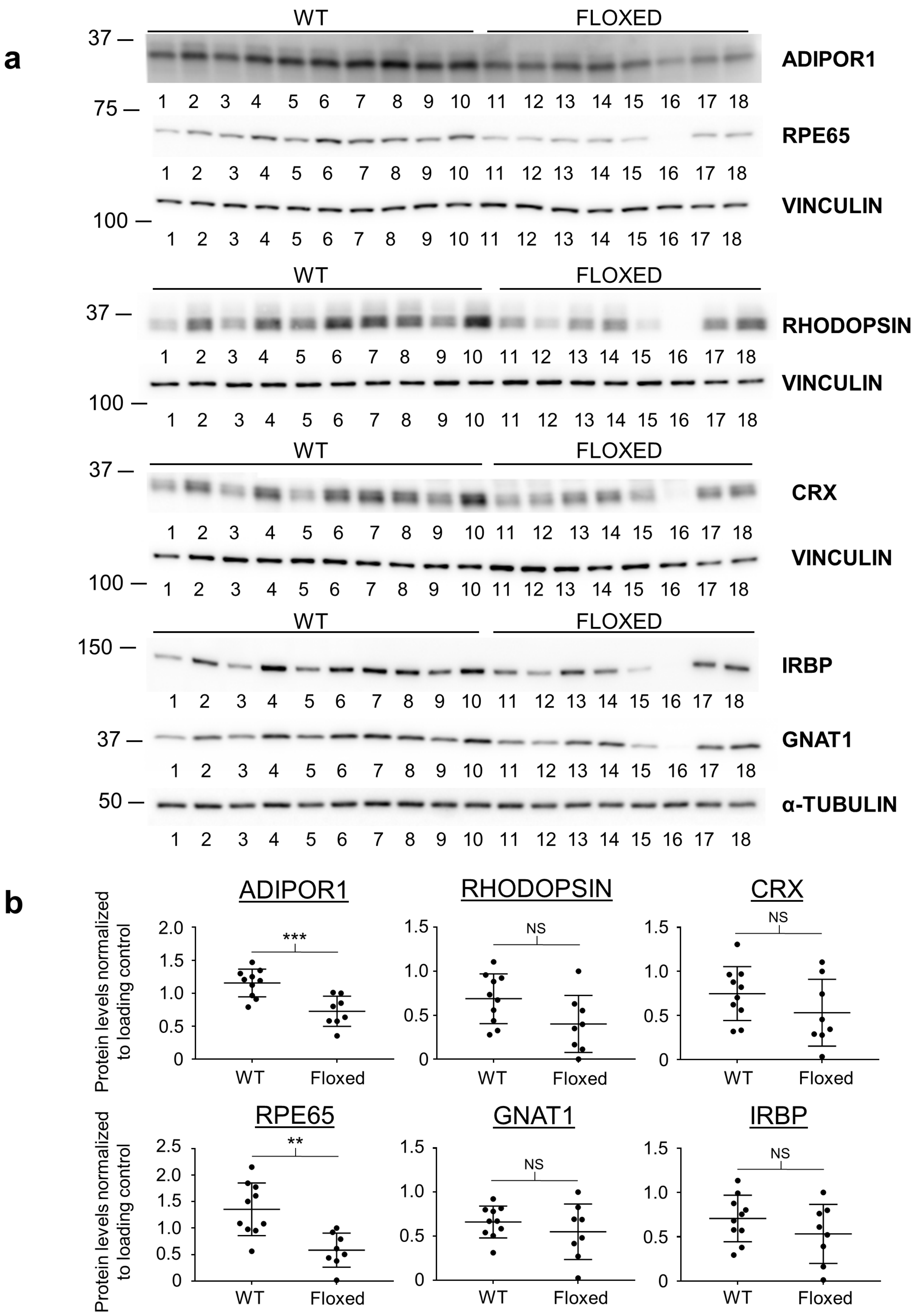

Supplementary Figure-8

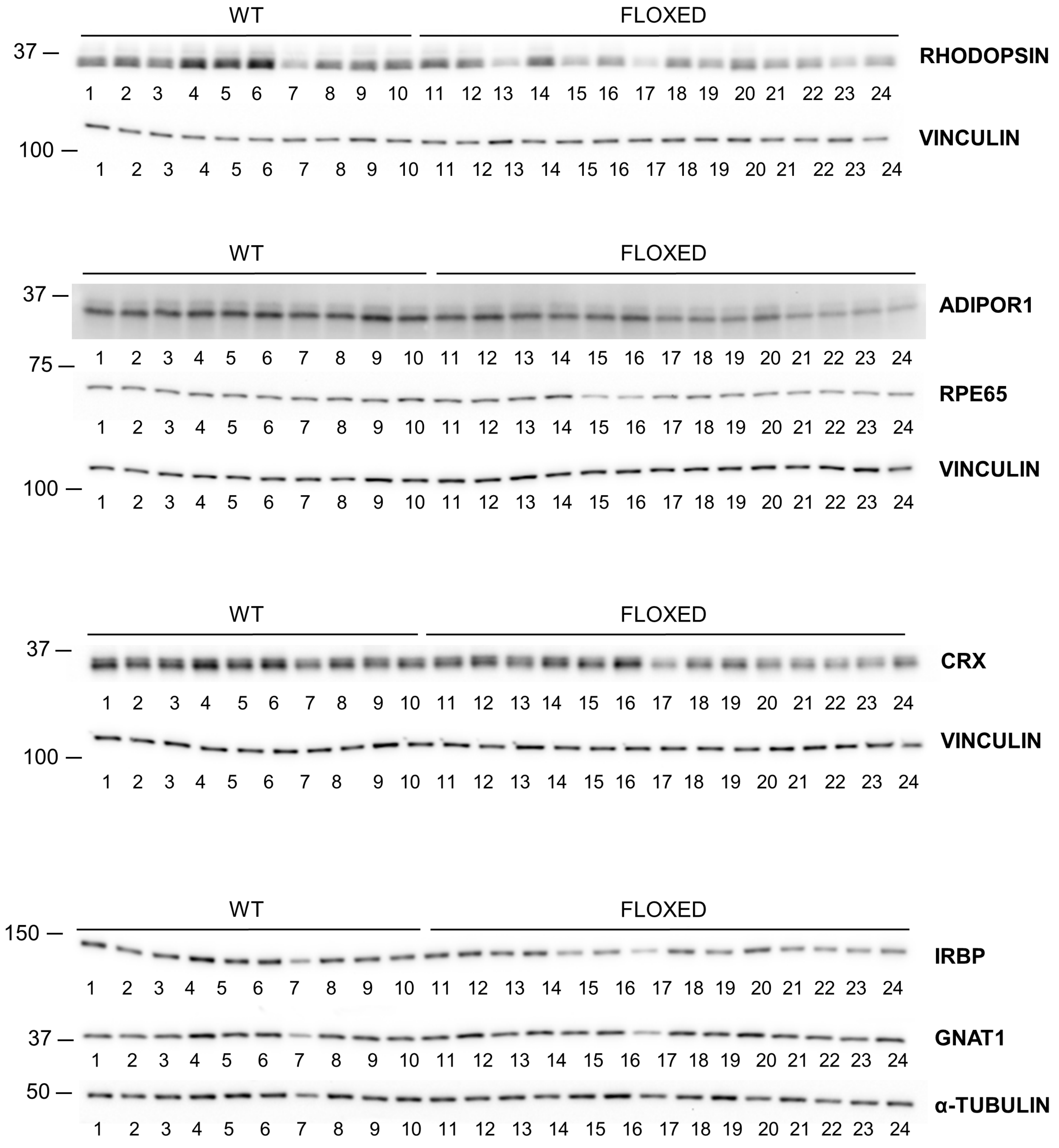

Supplementary Figure-9

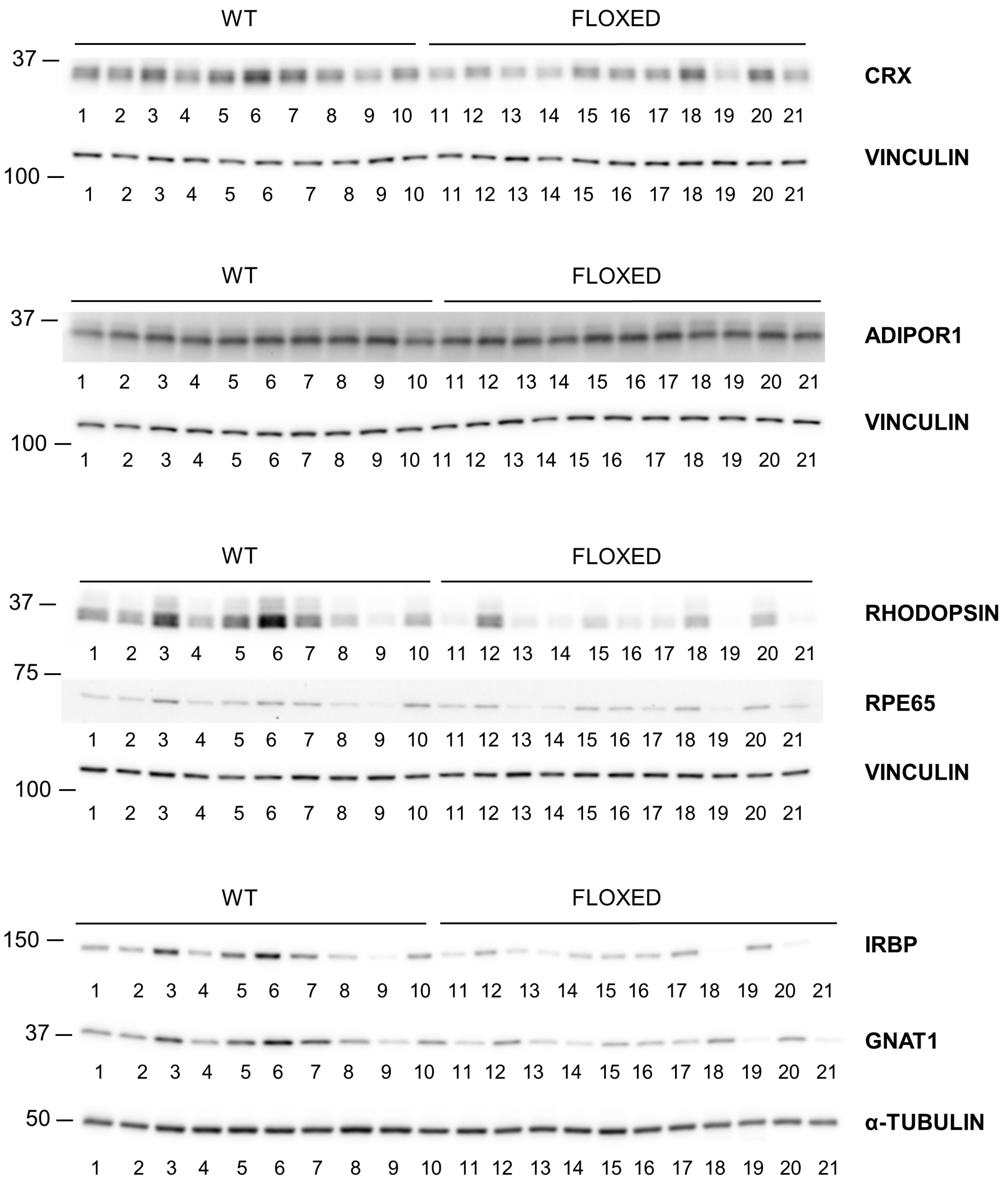

Supplementary Figure-10

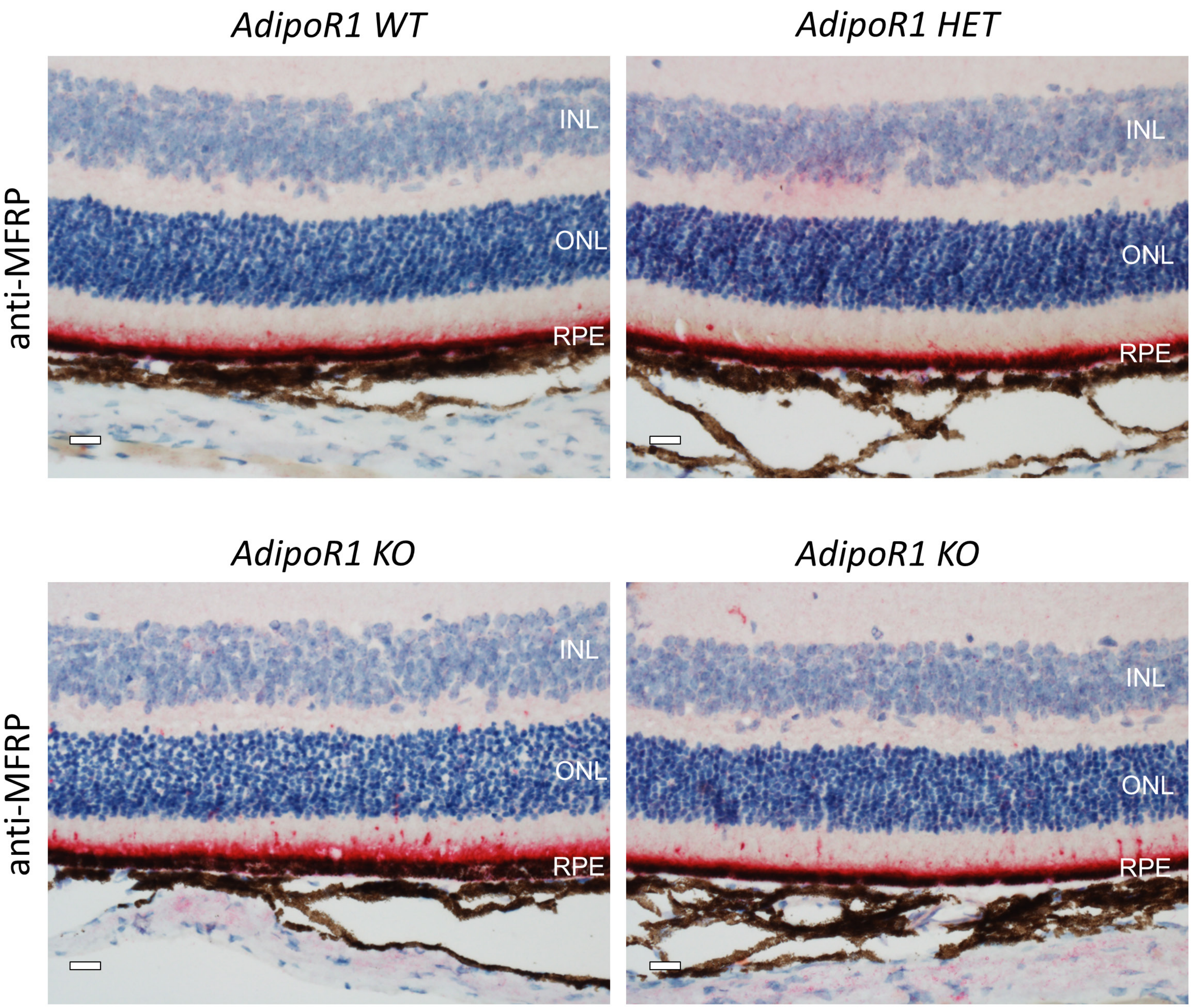

Supplement: Supplementary file 1 — Supplementary Information [file 41598_2018_32579_MOESM1_ESM.pdf]
